# Structural mechanisms of SLF1 interactions with Histone H4 and RAD18 at the stalled replication fork

**DOI:** 10.1093/nar/gkae831

**Published:** 2024-10-03

**Authors:** Emma L Ryder, Nazia Nasir, Amy E O Durgan, Michael Jenkyn-Bedford, Stephanie Tye, Xiaodong Zhang, Qian Wu

**Affiliations:** Astbury Centre for Structural Molecular Biology, School of Molecular & Cellular Biology, Faculty of Biological Sciences, University of Leeds, Leeds LS2 9JT, UK; Astbury Centre for Structural Molecular Biology, School of Molecular & Cellular Biology, Faculty of Biological Sciences, University of Leeds, Leeds LS2 9JT, UK; Astbury Centre for Structural Molecular Biology, School of Molecular & Cellular Biology, Faculty of Biological Sciences, University of Leeds, Leeds LS2 9JT, UK; Department of Biochemistry, University of Cambridge, 80 Tennis Court Road, CambridgeCB2 1GA, UK; Section of Structural and Synthetic Biology, Department of Infectious Disease, Imperial College London, London SW7 2AZ, UK; The Francis Crick Institute, 1 Midland Road, London NW1 1AT, UK; Section of Structural and Synthetic Biology, Department of Infectious Disease, Imperial College London, London SW7 2AZ, UK; The Francis Crick Institute, 1 Midland Road, London NW1 1AT, UK; Astbury Centre for Structural Molecular Biology, School of Molecular & Cellular Biology, Faculty of Biological Sciences, University of Leeds, Leeds LS2 9JT, UK

## Abstract

DNA damage that obstructs the replication machinery poses a significant threat to genome stability. Replication-coupled repair mechanisms safeguard stalled replication forks by coordinating proteins involved in the DNA damage response (DDR) and replication. SLF1 (SMC5–SMC6 complex localization factor 1) is crucial for facilitating the recruitment of the SMC5/6 complex to damage sites through interactions with SLF2, RAD18, and nucleosomes. However, the structural mechanisms of SLF1’s interactions are unclear. In this study, we determined the crystal structure of SLF1’s ankyrin repeat domain bound to an unmethylated histone H4 tail, illustrating how SLF1 reads nascent nucleosomes. Using structure-based mutagenesis, we confirmed a phosphorylation-dependent interaction necessary for a stable complex between SLF1’s tandem BRCA1 C-Terminal domain (tBRCT) and the phosphorylated C-terminal region (S442 and S444) of RAD18. We validated a functional role of conserved phosphate-binding residues in SLF1, and hydrophobic residues in RAD18 that are adjacent to phosphorylation sites, both of which contribute to the strong interaction. Interestingly, we discovered a DNA-binding property of this RAD18-binding interface, providing an additional domain of SLF1 to enhance binding to nucleosomes. Our results provide critical structural insights into SLF1’s interactions with post-replicative chromatin and phosphorylation-dependent DDR signalling, enhancing our understanding of SMC5/6 recruitment and/or activity during replication-coupled DNA repair.

## Graphical Abstract

**Graphical Abstract:**
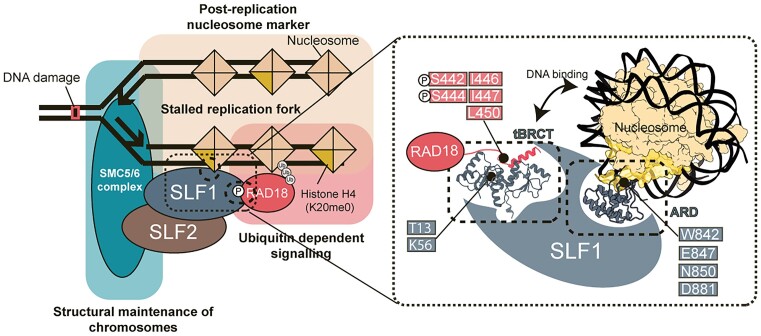

## Introduction

Genome integrity, the occurrence of cancer, and the effectiveness of cancer treatments are directly associated with the ability of our cells to sense and repair DNA damage through an orchestrated DNA damage response (DDR) network (1–3). DNA lesions, such as interstrand cross-links and DNA–protein crosslinks (DPCs), are particularly toxic during the S-phase of the cell cycle when these lesions block the progression of the replication machinery and cause replication fork stalling (4). This stalling can result in collapse of the replication fork, compromising DNA synthesis and increasing the risk of chromosomal aberrations, genome instability, and cell death (5). To counteract DNA damage encountered during replication, cells employ replication-coupled repair mechanisms that coordinate multiple pathways to safeguard the replication fork, overcome the obstructing lesions, and repair the DNA damage (5–7).

**Figure 1.**
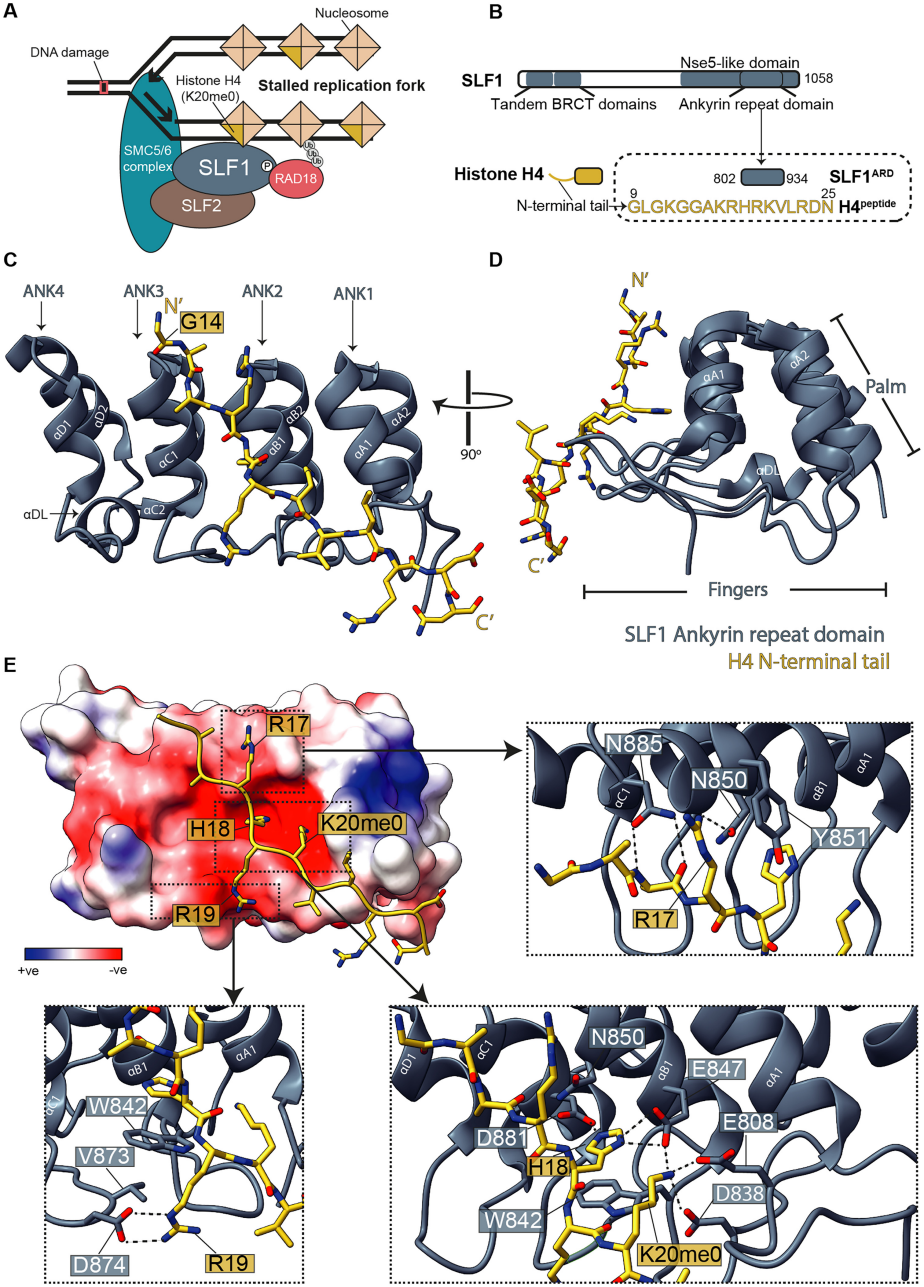
Crystal structure of SLF1^ARD^ bound to the unmethylated histone H4 tail. (**A**) Schematic illustrating the recruitment pathway of the SMC5/6 complex to stalled replication forks, involving RAD18, SLF1 and SLF2. Recruitment depends on nascent chromatin containing histone H4 unmethylated at lysine 20, RNF8–RNF168-mediated ubiquitination, and phosphorylation of RAD18 at S442 and S444. (**B**) Schematic highlighting the structural domains of human SLF1 and the N-terminal tail region of histone H4. The domain boundaries of the Ankyrin Repeat Domain (ARD) construct and the synthesized residues corresponding to the H4 tail peptide are indicated. (**C**) The 1.28 Å molecular model of SLF1^ARD^ (in grey) in complex with H4^peptide^ (in yellow). The ankyrin repeats (ANK1-4) of SLF1^ARD^ and individual helices are labelled. (**D**) Side-view of the structural model emphasizing the ‘finger’-like loop projections and the ‘palm’ formed by the ANK helices. (**E**) The electrostatic potential surface of SLF1^ARD^, revealing the concave acidic binding surface accommodating the H4 tail. Specific intermolecular interactions of H4 residues R17, H18, R19, and K20 (unmethylated) are detailed in the enlarged images corresponding to the indicated regions. Polar interactions are indicated by black dashed lines.

Recent advances in unbiased proteomics approaches and CRISPR-Cas9 genetic screens initiated a new wave of identifying proteins present at sites of stalling, revealing new insights into proteins whose roles within the DDR had been relatively uncharacterized (8–10). SMC5/6 localization factor 1 (SLF1), and its interaction partner SLF2, have been identified in multiple independent proteomic analyses as proteins that accumulate at stalled replication forks with early repair factors, as part of a RAD18-SLF1-SLF2-SMC5/6 genome stability pathway (11–14) (Figure 1A). Structural Maintenance of Chromosome (SMC) protein complexes, which include the SMC5/6 complex, cohesin and condensin, play an important role in regulating the higher-order chromosome structure. The ring-like organization of SMC complexes and an intrinsic ATPase motor facilitate the topological entrapment of DNA and loop extrusion, both of which play essential roles in DNA repair and promote efficient replication through difficult-to-replicate regions of the genome (15–21). SLF1 and SLF2 are the proposed human orthologs of yeast Nse5 and Nse6, which form a heterodimeric complex that serve as additional regulatory factors of the Smc5/6 complex (22–30). In yeast, Nse5-Nse6 mediates Smc5/6 chromatin recruitment and loading at DNA lesions and modulates the ATPase activity and DNA loop extrusion functions of the Smc5/6 complex. Consistent with this yeast phenotype, depletion of SLF1 or SLF2 has been shown to abrogate SMC5/6 recruitment to damage sites and sensitize cells to double-strand break (DSB)-inducing ionizing radiation and cross-linking agents (13). Furthermore, SMC5 and SLF2 mutations have been recently identified in patient-derived cells displaying a chromosomal instability phenotype (31). However, how SLF1 and SLF2 contribute to the regulation of SMC5/6 to maintain genome stability remains unclear, and the precise function of SLF1 remains undefined.

SLF1 is a 121 kDa protein containing a tandem BRCA1 C-terminal (tBRCT) domain at its N-terminus and a C-terminal Nse5-like protein domain split by an ankyrin repeat domain (ARD) (Figure 1B). Based on the high sequence similarity of this ARD to the ARD of two other DNA repair proteins, TONSL (Tonsoku-like protein) and BARD1 (BRCA1 associated RING Domain 1), the C-terminal ARD of SLF1 has been implicated in recognition of nucleosomes harbouring H4 unmethylated at K20, a characteristic of nascent nucleosomes incorporated during S phase (32–34). Consistently, pull-down experiments showed that SLF1 exhibits a clear preference for nucleosomes containing H4K20me0 over H4 mono- or di-methylated at K20 (32). However, while reported structures for the ARDs of TONSL and BARD1 in complex with a histone H3–H4 dimer (33) or nucleosome core particle (35,36), respectively, reveal how the ARD of these proteins mediate binding to nascent nucleosomes, there is currently no structural evidence for H4K20me0 recognition by SLF1.

Prior to its identification as part of a RAD18–SLF1–SLF2–SMC5/6 complex, earlier studies noted an interaction between SLF1 and the C-terminus of RAD18, establishing a role of SLF1 as a DNA damage response protein (37). This function occurs downstream of ubiquitin-dependent DDR signalling mediated by MDC1 and RNF8/RNF168, as well as RAD18, in response to UV-induced DNA damage. RAD18 is a highly conserved E3 ubiquitin ligase that coordinates multiple DNA repair and damage tolerance pathways (38–41). The interaction between RAD18 and SLF1 is mediated by the N-terminal tBRCT of SLF1 and requires the phosphorylation of RAD18 residues S442 and S444 (13,37). Notably, this interaction was shown to be independent of RAD18’s association with the ubiquitin-conjugating enzyme (E2) RAD6, and dispensable for RAD18’s well established functions in PCNA mono-ubiquitination and homologous recombination (HR) (37,38,42–46). Hence, RAD18 appears to serve a noncatalytic scaffolding role for SLF1 in response to stalled replication forks. Recent structural analysis sheds light on how SLF1’s tBRCT recognizes a phosphorylated RAD18 peptide, employing a mechanism common among tandem BRCT domains (47). However, additional functions or roles of this domain within the DDR remain to be explored.

In this study, we have determined the crystal structure for the ARD of SLF1 in complex with an unmodified histone H4 peptide (K20me0) at 1.28 Å resolution. Additionally, we have characterized key residues in SLF1 that are involved in interacting with nascent nucleosomes, allowing for a comparison of the conserved mechanism of H4K20me0 recognition across three highly conserved nascent nucleosome reader proteins. Using an AlphaFold2 (AF2) model and conducting mutagenesis studies, our findings validate the recently determined structural basis for the interaction between the tBRCT of SLF1 and the C-terminus of RAD18 and offer further insights into the specificity requirements of this interaction. Furthermore, we have identified a high-affinity DNA-binding property of the SLF1 tBRCT, which shares an interaction surface with the RAD18 binding site. Together with the histone-binding property of the ARD, this DNA-binding property of tBRCT further contributes to the interaction between SLF1 and the nucleosome. Our study reveals an intriguing interplay between tBRCT binding with the RAD18 C-terminus and DNA, providing novel insights into the functional role of this domain within SLF1.

## Materials and methods

### Construction of recombinant plasmids

The cDNA for full-length human SLF1 and RAD18 were synthesized (GenScript). The DNA for residues 6–208 (SLF1^tBRCT^) or 802–934 (SLF1^ARD^) of SLF1 were amplified and subcloned into pHAT4 (48) (N-terminal cleavable His-tag). A C-terminal RAD18 construct (RAD18^CT^) was produced by subcloning DNA for residues 438–495 into pMAT11(48) (N-terminal cleavable His-MBP-tag). Point mutations were introduced by site-directed mutagenesis (SDM) polymerase chain reaction (PCR) using Q5® Site-Directed mutagenesis kit (NEB) and confirmed by DNA sequencing. These mutant constructs include SLF1^ARD_4A^ (with the four mutations W842A, E847A, N850A and D881A), SLF1^tBRCT_T13A^, SLF1^tBRCT_K56A^, RAD18^CT_S442D/S444D^, RAD18^CT_S442D/S444D/I446K^, RAD18^CT_S442D/S444D/I447A^, RAD18^CT_S442D/S444D/L450K^, and RAD18^CT_S442D/S444D/L451K^. A BRCA1^tBRCT^ (residues 1646–1859) construct in pHAT2 (48) (N-terminal non-cleavable His-tag), was the same as used for a previous study (49).

### Recombinant protein expression and purification of SLF1, RAD18, BRCA1 and nucleosome

All wild-type and mutant constructs were transformed into *Escherichia coli* strain BL21 (DE3) competent cells (C2527, NEB). The expression of SLF1 constructs was induced using 1 mM IPTG at 16°C overnight. RAD18 constructs were expressed using 0.25 mM IPTG at 37°C for 3 h. SLF1 cells were harvested and resuspended in wash buffer (50 mM Tris pH 8.0, 500 mM NaCl, 20 mM imidazole, 1 mM TCEP), supplemented with 1 mM AEBSF hydrochloride. Cells were lysed by sonication, then clarified by centrifugation (30 000 × g, 30 mins, 4°C). The clarified supernatant was incubated with Nickel-Sepharose® 6 fast flow beads (Cytiva), washed with wash buffer, and eluted in elution buffer (50 mM Tris pH 8.0, 300 mM imidazole, 500 mM NaCl, 1 mM TCEP). For pull-down experiments, the His-tags of SLF1^ARD^ and SLF1^ARD_4A^ proteins were retained. For His-tag cleavage for all other experiments, cleavage was performed overnight at 4°C using TEV protease, with concurrent dialysis (20 mM Tris pH 8.0, 300 mM NaCl, 1 mM TCEP). After incubation with Nickel-Sepharose® 6 fast flow beads, the flow through was collected and concentrated for size-exclusion chromatography (SEC) using a Superdex 75 Increase 10/300 column (GE Life Science) in gel filtration buffer (20 mM Tris pH 8.0, 150 mM NaCl, 1 mM TCEP). RAD18 proteins were purified by Ni affinity as described for SLF1 proteins, except using HEPES (pH 7.5) instead of Tris. The tags of RAD18^CT^ were retained. For the SEC of RAD18, 10% glycerol was added to the purification buffers, and a Superdex 200 Increase 10/300 column was used. All elution samples were analysed using NuPAGE 4–12% Bis–Tris gels (Invitrogen), which were stained using InstantBlue® Coomassie Protein Stain (Abcam) and imaged using ChemiDoc XRS+ (Bio-Rad). Fractions containing pure samples were pooled and concentrated. Protein concentration was measured using Implen nanophotometer NP80. Final protein samples were flash frozen in liquid N_2_ and stored at –80°C. BRCA1^tBRCT^ was expressed and purified as described in a previous study (49).

Human histones H2A and H2B were co-expressed using a modified pCDF vector in *E. coli*. Cells were lysed by sonication in 20 mM Tris pH 7.5, 500 mM NaCl, 0.1 mM EDTA, 1 mM TCEP, and 2 Roche protease inhibitor tablets per 100 ml lysis buffer. H2A and H2B were purified as soluble dimers on HiTrap Q FF and HiTrap Heparin HP in buffer A (20 mM Tris pH 7.5, 500 mM NaCl, 1 mM EDTA, 1 mM TCEP), and eluted by a salt gradient with buffer B (20 mM Tris pH 7.5, 2 M NaCl, 1 mM EDTA, 1 mM TCEP), followed by gel filtration with Superdex 200 10/300 in buffer B. Human histones H3.1 and H4 were co-expressed from a pETDuet vector in *E. coli*, lysed by sonication and purified as soluble tetramers on HiTrap Heparin HP in buffer A and eluted by a salt gradient with buffer B, followed by Superdex 200 16/600 in buffer B. Nucleosomes were reconstituted by salt gradient dialysis of the H2A–H2B dimer, H3.1–H4 tetramer, and a 147 bp DNA fragment based on the Widom 601 positioning sequence (50).

All purified protein samples used in this study are shown in Supplementary Figure S1 or Figure 3B.

### Synthesized peptides for RAD18 and H4

All peptides were synthesized above 95% purity with modifications at both N and C terminus (Biomatik). The histone H4 peptide contains N-terminal biotinylation and C-terminal amidation. All RAD18 peptides contain N-terminal acetylation and C-terminal amidation. All peptides were dissolved in 100 mM Tris pH 8.0, 150 mM NaCl, 1 mM TCEP (RAD18 peptides) or 20 mM Tris pH 8.0, 150 mM NaCl buffer (H4 peptides) and stored at –80°C. The names and sequences of all peptides are: H4^peptide^: GLGKGGAKRHRKVLRDN; H4^K20me1_peptide^: GLGKGGAK(me1)RHRKVLRDN; H4^K20me2_peptide^: GLGKGGAK(me2)RHRKVLRDN; RAD18^peptide^: SSSSDI; RAD18(442p)^peptide^: S(pS)SSDI; RAD18(444p)^peptide^: SSS(pS)DI; RAD18(442p,444p)^peptide^: S(pS)S(pS)DI; RAD18(442p,444p,I446A)^peptide^: S(pS)S(pS)DA; RAD18(442p,444p,I446K)^peptide^ : S(pS)S(pS)DK, 6-FAM-RAD18(442p,444p)^peptide^ : 6-FAM-S(pS)S(pS)DI (where 6-FAM is 6-Carboxyfluorescein, p indicates phosphorylation, me indicates methylation).

### Protein crystallization, data collection and structural determination

Purified SLF1^ARD^ was incubated with H4^peptide^ in a 1:2 molar ratio at 4°C for 1 h. The final protein concentration was 20 mg/ml. Crystallization employed a sitting-drop vapour diffusion method by using the mosquito® HTS Nanolitre Liquid Handler system (SPT Labtech, UK) to prepare drops containing 0.15 μl of protein sample and 0.15 μl of crystallization solution. The complex crystallized at 18°C after two weeks in a condition containing 0.1 M sodium malonate dibasic monohydrate, 0.1 M HEPES pH 7, 30% w/v poly(acrylic acid sodium salt) 2100. Diffraction data was collected remotely at the I24 beamline at the Diamond Light Source. The crystal diffracted to 1.28 Å resolution. Data sets were processed using the autoprocessing pipeline xia2 3dii (51) at the Diamond Light source. The Phenix software suite (v1.20.1-4487) was used for structural determination (52). Phases were obtained by molecular replacement in Phaser (53) using a Phyre2-generated homology model (54) for SLF1^ARD^ as the search model. Coot and Phenix were used for model building and refinement following standard refinement strategy (52,55). The last residue of SLF1^ARD^ and the first five residues of the H4 peptide were not resolved due to flexibility. The remaining amino acids could be reliably fit into the density. The buried surface area within SLD1^ARD^-H4^peptide^ was calculated using PISA (56).

### Circular dichroism

Circular dichroism spectra for wild-type, and mutants of SLF1^ARD^ and SLF1^tBRCT^, in 10 mM NaH_2_PO_4_ (pH 7.0), were recorded and analysed using a Chirascan™ plus circular dichroism spectrometer (Applied Photophysics) between 180 and 260 nm in a 1 mm path-length cuvette, with three accumulations, 2 nm bandwidth, and a scanning speed of 60 nm/min at 20°C. Results were plotted by GraphPad Prism v9.4.0.

### Pull-down assay

The H4 peptides, H4^peptide^, H4^K20me1_peptide^ or H4^K20me2_peptide^, were incubated with His-tagged SLF1^ARD^ or SLF1^ARD_4A^ in a 20 μl total volume for 1 h at 4°C using the indicated concentrations. Each reaction was incubated with 30 μl Dynabeads MyOne Streptavidin T1 (ThermoFisher Scientific), pre-blocked in 2X Casein blocking buffer (Sigma Aldrich) diluted in assay buffer (20 mM Tris pH 8.0, 150 mM NaCl) for 1 h at 4°C. The beads were then washed 4X with 500 μl assay buffer. After the last wash, the beads were boiled in SDS-PAGE sample buffer and analysed using NuPAGE 4–12% Bis–Tris gels (Invitrogen) and InstantBlue® Coomassie Protein Stain.

### Electrophoretic mobility shift assay

For EMSA with double-stranded DNA, SLF1^ARD^, SLF1^tBRCT^, or BRCA1^BRCTs^ were incubated with 10 nM 50 bp 6-FAM labelled DNA in DNA binding buffer (40 mM Tris pH 7.5, 100 mM KCl, 0.2 mM DTT, 10% glycerol, 20 μg/ml bovine serum albumin) to a final volume of 10 μl at 4°C for 30 mins, using the protein concentrations indicated. For competitive EMSA, 10 nM 50 bp DNA and 0.5 μM SLF1^tBRCT^ were incubated with the indicated concentrations of RAD18 peptides. The 50 bp DNA substrate was prepared by annealing the primers 5′ - [6-FAM]-TAAATGCCAATGCTGCTGATACGTACTCGGACTGATTCGGAACTGTAACG-3′ and 5′-CGTTACAGTTCCGAATCAGTCCGAGTACGTATCAGCAGCATTGGCATTTA-3′ by incubation at 95°C for 2 mins then cooling to 25°C over 40 mins. EMSA reaction samples were resolved by non-denaturing polyacrylamide gel electrophoresis (PAGE) (0.5X TBE buffer, 100 V, 65 mins, 4°C) using Novex 6% DNA retardation gels (ThermoFisher). Gels were imaged using the iBright™ FL1500 imaging system (Invitrogen).

Nucleosome EMSAs were performed by incubating SLF1^tBRCT^, SLF1^ARD^ or SLF1^ARD_4A^ with 10 nM nucleosome core particles at room temperature for 20 mins. Nucleosome position was observed using Diamond DNA stain (Promega), diluted 1:10 000 in 0.5X TBE. To test for simultaneous binding of SLF1^tBRCT^ and SLF1^ARD^, 1 μM SLF1^tBRCT^ was incubated with 10 nM nucleosome core particle and the concentrations of SLF1^ARD^ indicated.

### Analytical size exclusion chromatography

SLF1^ARD^ and SLF1^tBRCT^ were incubated in a 2:1 molar ratio using a final concentration of 20 and 10 μM, respectively. Wild-type or mutant SLF1^tBRCT^ were incubated with RAD18^CT^ proteins in a 1:1 molar ratio using a final protein concentration of 10 μM. All samples were prepared to a final volume of 200 μl and incubated on ice for 60 mins. Gel filtration was performed with 20 mM Tris pH 8.0 and 150 mM NaCl using a Superdex 75 Increase 10/300 or Superdex 200 Increase 10/300 column (GE Healthcare) with a flow rate of 0.5 ml/min. Eluted fractions were analysed using NuPAGE 4–12% Bis–Tris gels (Invitrogen).

### Thermal melt

Protein thermal stability was measured within 100 μl reactions containing 10 μM SLF1^tBRCT^ proteins, 200 μM RAD18 peptides, and 5X (working concentration) SYPRO® Orange protein gel stain (ThermoFisher). SLF1^tBRCT^ thermal stability in the presence of DNA was determined using 30 μM 50 bp DNA. The 50 bp DNA substrate was prepared by annealing the primers 5′ -TAAATGCCAATGCTGCTGATACGTACTCGGACTGATTCGGAACTGTAACG-3′ and 5′-CGTTACAGTTCCGAATCAGTCCGAGTACGTATCAGCAGCATTGGCATTTA-3′, as described above. Fluorescence was monitored from each reaction in triplicate at a wavelength of 568 nm using a QuantStudio™ 3 Real-Time PCR machine, incubating at 25°C for 10 mins, followed by a temperature gradient from 25–95°C in 30 s steps of 0.5°C. Average melting temperature values from three replicate wells were determined from the derivative curves using the Protein Thermal Shift Software v1.4. The experiment was repeated in triplicate and plotted in GraphPad Prism v10.1.0. Statistical analysis was performed using a Brown-Forsythe and Welch one-way analysis of variance (ANOVA) in GraphPad, using a Dunnett T3 test to compare the mean *T*_m_ of each SLF1^tBRCT^ protein with each peptide to the mean of the respective SLF1^tBRCT^ only as the post-hoc test. A two-tailed t-test was used to test for statistical significance for the *T*_m_ of each SLF1^tBRCT^ protein with or without DNA. Statistical significance in the data was assessed using *P* values as follows: ns *P* > 0.05, *P ≤ 0.05, **P ≤ 0.01, ***P ≤ 0.001, ****P ≤ 0.0001.

### Fluorescence polarization

10 nM 6-FAM labelled 50 bp DNA, or 20 nM 6-FAM-RAD18(442p,444p)^peptide^ was incubated at room temperature for 30 mins with the indicated concentrations of SLF1^tBRCT^ or SLF1^tBRCT_K56A^ in 20 mM Tris pH 8.0, 150 mM NaCl, and 5% glycerol. Fluorescence polarization was measured using a Hidex Sense microplate reader (Hidex) with an excitation wavelength of 485 nm and detection of emission at 520 nm. The difference in polarization signal relative to the fluorophore alone was first calculated. This difference was then fitted using a one-site specific binding model (*Y* = *B*_max_*X/(*K*_d_+ *X*)). Data represent the mean of three separate experiments. Results were plotted using GraphPad Prism v10.1.0.

### Structure prediction, analysis, and presentation

Predicted structures of individual proteins were obtained from the AlphaFold Protein Structure Database (57). ColabFold v1.5.2-patch was used to generate the AlphaFold2-predicted complex structure of SLF1^tBRCT^-RAD18^CT_S442D_S444D^, using default settings (58). Structure superimposition of tandem BRCT domains from SLF1 (AFDB accession: AF-Q9BQI6-F1), BRCA1 (PDB:1JNX) (59), BARD1 (PDB: 2NTE) (60); MCPH1 (PDB:3SHT) (61), MDC1 (PDB: 2ADO) (62), PTIP (PDB:3SQD) (63), TOPBP1 (PDB: 3AL2) (64), 53BP1 (PDB: 5ECG) (65), and Nibrin (AFDB accession: AF-O60934-F1) was performed using ChimeraX (v1.4) (66). A structure-based sequence alignment from the resulting superimposition was generated in Chimera (67). The sequence conservation was then displayed graphically on the AF2 SLF1^tBRCT^ structure.

AlphaFold3 (AF3) predicted complexes were generated using the AF3 server with default settings (68). The predicted complex of 50 bp DNA with SLF1^tBRCT^ was generated using the DNA sequence for the substrate used in EMSA experiments. The predicted complex of SLF1 bound to the nucleosome was generated using the sequences of histone H2A2A (UniProt ID: Q6FI13), H2B1B (UniProt ID: P33778), H3.3C (UniProt ID: Q6NXT2), H4 (UniProt ID: P62805), SLF1 (UniProt ID: Q9BQI6), and the 147 bp 601 Widom DNA sequence (50).

Electrostatic potential and all structure figures were generated using ChimeraX (v1.4) (66).

### Multiple sequence alignment

Multiple sequence alignments were performed using Clustal Omega (69).

## Results

### Crystal structure of the SLF1 ankyrin repeat domain bound to histone H4 tail containing unmethylated K20 (K20me0)

To experimentally validate the interaction between SLF1 and H4 at the atomic level, we have crystallized the SLF1-H4 complex, comprising the recombinantly expressed ARD of SLF1 (SLF1^ARD^, residues 802-934) and a synthetic peptide of H4 N-terminal tail (H4^peptide^, residues 9-25) (Figure 1 and Supplementary Figure S2A, B). The crystal structure of SLF1^ARD^-H4^peptide^ was determined by molecular replacement at 1.28 Å resolution (Table 1), revealing the structure of SLF1^ARD^ and its interface with H4. SLF1^ARD^ contains four ankyrin repeats (labelled as ANK1-4 in Figure 1C and D), each containing a typical helix-turn-helix fold, with loops connecting consecutive repeats. The overall structure of SLF1^ARD^ is concave in shape, like a ‘cupped hand’, as described for many other ARD structures (70–73). The well-defined extended β loop structures form the ‘fingers’ (labelled Finger 0-3, Supplementary Figure S2B), and four pairs of stacking helices form the ‘palm’ (Figure 1D). The inner surface, formed by the first helices from each ANK (labelled as αA1, αB1, αC1, and αD1 in Figure 1C and Supplementary Figure S2B), creates a negatively charged binding cavity. The H4 peptide is held by this ‘cupped hand’ with a calculated interaction surface area of 589 Å^2^, predominantly involving ANK1-3.

**Table 1.** Data collection and refinement statistics of the SLF1^ARD^-H4^peptide^ crystal structure

**Crystal**	SLF1^ARD^-H4^peptide^
**Data collection**	
X-ray source	Diamond Beamline i24
Wavelength (Å)	0.99
Space group	C 2 2 21
Cell dimensions *a*, *b*, *c* (Å) α, β, γ (°)	37.68, 50.94, 139.62, 90, 90 and 90
Resolution (Å)	27.79–1.28 (1.33–1.28)
*R*_merge_^a^	0.12 (1.86)
Mean I/sigma(I)	10.02 (1.38)
Completeness (%)	99.97 (100.00)
Redundancy	11.40 (8.50)
CC1/2	0.99 (0.33)
Wilson B factor (Å^2^)	17.09
**Refinement**	
Resolution (Å)	27.79–1.28 (1.33–1.28)
No. of unique reflections	35 136 (3460)
No. of unique reflections used for R-free	1763 (178)
*R*_work_^b^	0.19 (0.39)
*R*_free_^c^	0.23 (0.40)
No. non-hydrogen atoms	1228
Protein	1113
Water	115
No. copy number of complex in ASU	1
*B*-factors (Å^2^)	25.30
Protein	24.11
Water	36.80
Ramachandran favoured (%)	99.3
Ramachandran allowed (%)	0.7
Ramachandran outliers (%)	0
Rotamer outliers (%)	0.82
Clashscore	1.78
R.m.s. deviations	
Bond lengths (Å)	0.005
Bond angles (°)	0.77

Statistics for the highest-resolution shell are shown in parentheses.

^a^ *R*_merge_ = Σ_h_|*I*_h_ − |/Σ_h_*I*_h_, where *I*_h_ is the intensity of reflection h, and is the mean intensity of all symmetry-related reflections.

^b^ *R*_work_ = Σ||*F*_obs_| −|*F*_calc_||/Σ|*F*_obs_|, *F*_obs_ and *F*_calc_ are observed and calculated structure factor amplitudes.

^c^ *R*_free_ as for *R*_work_ using a randomly selected 5% of SLF1^ARD^-H4^peptide^ data excluded from the refinement.

**Figure 2.**
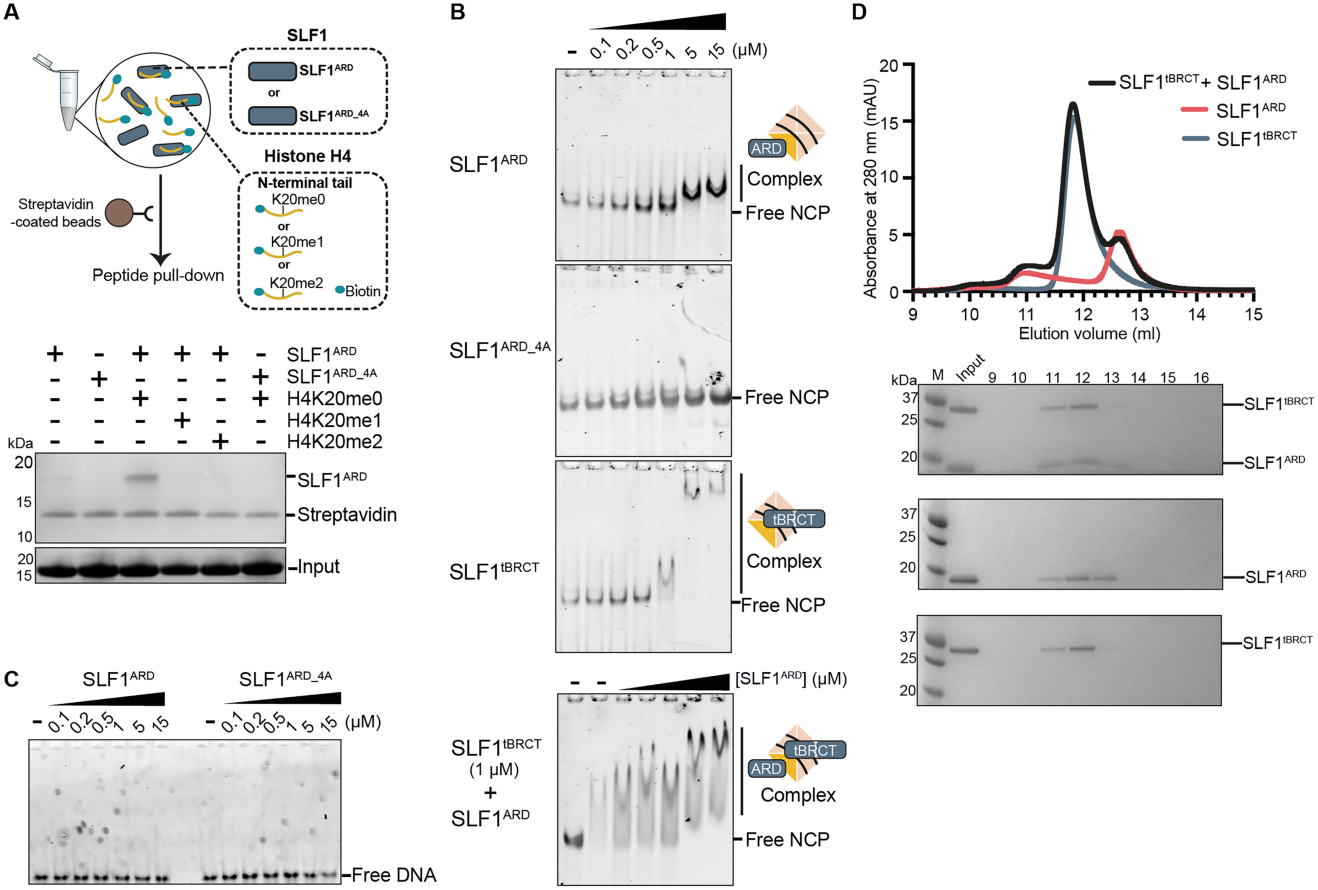
The tandem BRCT and ARD of SLF1 recognize separate features of nascent nucleosomes. (**A**) Pull-down of purified SLF1^ARD^ proteins (WT or 4A mutant) using biotinylated H4 peptides, with or without methylation at lysine 20. Pulled down proteins were analysed by Coomassie staining, with input samples presented in the bottom panel. The protein band corresponding to streptavidin serves as a control, indicating equal amounts of streptavidin-coated beads were used. A schematic illustrating the experimental set-up of the pull-down experiments performed is shown above. (**B**) Representative electrophoretic mobility shift assay (EMSA) gels for the interaction of WT or 4A mutant SLF1^ARD^, SLF1^tBRCT^, or SLF1^tBRCT^ and SLF1^ARD^, with unmodified nucleosome core particles (NCPs) (representative of at least 2 replicates). Each lane contains 10 nM NCP, with the concentration of SLF1 protein indicated. For the EMSA with SLF1^tBRCT^ and SLF1^ARD^ (bottom panel) lanes 2 to 7 contained 1 μM SLF1^tBRCT^. Lanes 3-7 additionally contain SLF1^ARD^ at the indicated concentrations (0.2-15 μM). (**C**) Representative electrophoretic mobility shift assay (EMSA) gel for the interaction of WT or 4A mutant SLF1^ARD^ with 6-FAM labelled 50 bp DNA (representative of 3 replicates). Each lane contains 10 nM DNA substrate, with the concentration of SLF1 protein indicated. (**D**) Representative trace for UV absorbance recorded for analytical size exclusion chromatography for SLF1^ARD^ and SLF1^tBRCT^ (representative of three repeats). Coomassie-stained analysis of the elution fractions is shown beneath the chromatogram.

In our crystal structure, the electron density for H4 residues 9-13 and the side chain of K16 are not observed due to structural flexibility. SLF1^ARD^ interacts mainly with H4 residues R17, H18, R19, and K20me0 (Figure 1E). R17 mediates a polar interaction with N850^SLF1^ (Figure 1E, right top), while H18 and K20me0 bind within an acidic cavity formed by SLF1 residues D881, N850, E847, W842, D838 and E808, with H18 forming a π-π stacking interaction with W842^SLF1^ and a hydrogen bond with D881^SLF1^ (Figure 1E, right bottom). R19 forms a hydrogen bond with the indole ring of W842^SLF1^ and a salt bridge with D874^SLF1^ outside the central binding cavity (Figure 1E, left bottom). Unmethylated H4 K20 (H4K20me0) is an essential histone marker in post-replicative chromatin recognized by SLF1^ARD^ (32). Importantly, K20me0 facilitates a stable interaction with SLF1 by forming salt bridges with D838, E808, and E847, explaining why unmethylated K20 is critical for recruiting SLF1 to nucleosomes (Figure 1E, right bottom). This specificity was validated using pull-down experiments with N-terminal biotinylated H4 peptides, demonstrating that mono- or di-methylation at K20 abrogates the interaction observed with the unmethylated peptide (Figure 2A).

With our SLF1^ARD^-H4^peptide^ structure, we can now compare the similarities and differences between the ARDs of SLF1, TONSL, and BARD1, and how H4 interacts with them individually (33,35,36). The ARD in all these proteins contains four ANKs, with ANK1-3 exhibiting a canonical structure, while ANK4 is a C-terminal capping repeat. All three ARDs contain highly conserved residues that form the ankyrin repeat consensus sequence important for the tertiary fold of the repeats (73). Residues that form the acidic binding cavity across ANK1-3 are also conserved across the three proteins (Supplementary Figure S2C, D). Overlaying the structure of the ARD from SLF1, BARD1, and TONSL shows identical positioning of the side chains of these conserved residues, indicative of a conserved recognition surface tailored for H4K20me0 binding (Supplementary Figure S2E).

The most noticeable differences among SLF1, TONSL, and BARD1 are the size of ANK4, and the loop of Finger 2 connecting ANK2 and 3 (Supplementary Figure S2C, E). In this loop, a different side chain contacts the H4 R19 side chain. SLF1^ARD^ contains the shortest ANK4 helices, while TONSL^ARD^ contains the longest ANK4 and the longest Finger 2. The elongated helices of TONSL ANK4 appear to facilitate additional interactions with residues 12 and 13 of the H4 tail, which were not observed in our structure. We also observe an additional helix between ANK3 and 4 of SLF1^ARD^ (residues 909-932) (labelled as αDL in Figure 1C and Supplementary Figure S2A and C) formed within the ‘loop’ region leading to Finger 3.

### SLF1^ARD_4A^ mutant displays reduced affinity for the H4 N-terminal tail in nucleosome binding

To validate the observations from our crystal structure, as well as previously reported findings (32), we generated the SLF1^ARD_4A^ mutant, by mutating four residues (W842A, E847A, N850A, and D881A) involved in key electrostatic and hydrophobic interactions with the basic region of the H4 N-terminal tail. Subsequent pull-down assays with this mutant demonstrated a loss of binding affinity towards the H4 tail compared to wild-type SLF1^ARD^ (Figure 2A). Far-UV circular dichroism spectroscopy indicates that mutations in SLF1^ARD_4A^ did not affect the overall fold of the ARD itself (Supplementary Figure S3A), affirming that these four key residues (W842, E847, N850, and D881) play an important role in the direct interaction between SLF1^ARD^ and the H4 tail.

To validate that SLF1^ARD^ itself can interact with unmodified H4 in the nucleosome context, electrophoretic mobility shift assays (EMSA) were carried out using recombinantly reconstituted nucleosome core particles (NCPs) containing no post-translational modifications. Our results show an obvious slower migration of NCPs in the presence of 5 μM SLF1^ARD^, suggesting that the purified SLF1^ARD^ domain can interact with NCPs with an affinity of 1-5 μM (Figure 2B). In comparison, no change in nucleosome migration was observed for SLF1^ARD_4A^ using the same concentration range. This apparent reduction in affinity indicates that nucleosome binding of SLF1^ARD^ is notably abrogated by mutation of the acidic H4K20me0 binding pocket. We also observed no shift in the migration of fluorescently labelled 50 bp DNA by SLF1^ARD^ using EMSA experiments (Figure 2C). Together, these results indicate that the SLF1^ARD^ is sufficient to interact with unmodified nucleosomes, and that its mechanism of nucleosome binding primarily involves the recognition of the unmodified H4 N-terminal tail. It should be noted that we also performed experiments using up to 50 μM of SLF1^ARD_4A^ with unmodified nucleosomes. Here we observed a low-affinity interaction, suggesting that there may be some additional contributions from interactions involving residues outside of the H4 binding pocket (Supplementary Figure S4).

### The double phosphorylated C-terminus of RAD18 stabilizes the tandem BRCT domains of SLF1 in solution

**Figure 3.**
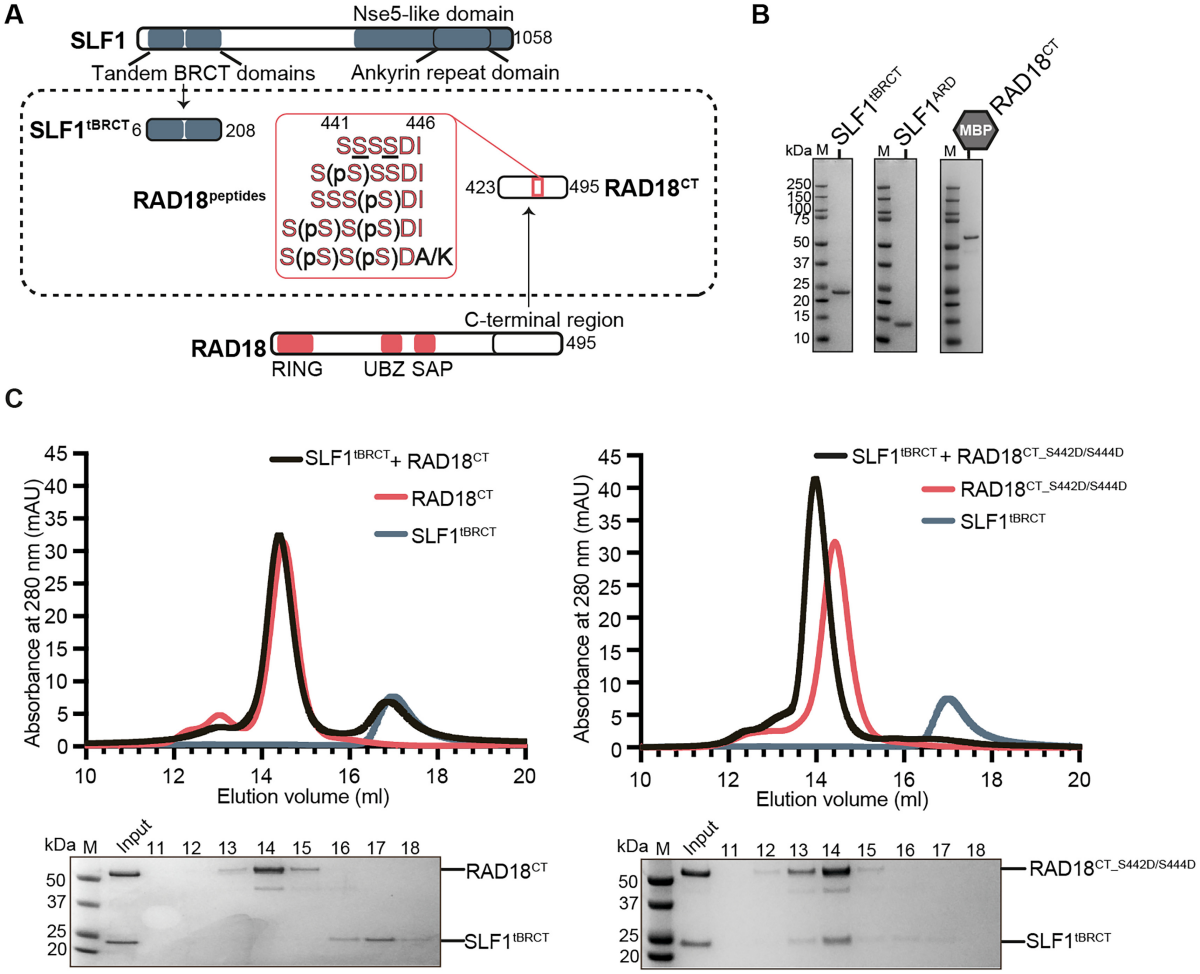
The double phosphorylated C-terminus of RAD18 stabilizes the tandem BRCT domains of SLF1 in solution. (**A**) Schematic highlighting the structural domains of human SLF1, as in Figure 1, and human RAD18. The domain boundaries of the Ankyrin Repeat Domain (SLF1^ARD^), tandem BRCT (SLF1^tBRCT^), and C-terminal RAD18 (RAD18^CT^) construct used in this study are indicated, along with the sequences of chemically synthesized RAD18 peptides spanning the critical phosphorylation sites (underlined). (**B**) Coomassie-stained analysis of 2 μg of purified domains used in subsequent experiments, for size reference. Analysis of all purified proteins can be found in Supplementary Figure S1. The purified RAD18^CT^ protein retained an N-terminal His-MBP tag. (**C**) Gel filtration for SLF1^tBRCT^ with WT (left) or phosphomimetic (S442D/S444D) RAD18^CT^ (right). Coomassie-stained analysis of elution fractions are shown beneath each chromatogram. Data are representative of at least two repeats.

Previous studies using cellular pull-down experiments have concluded that the SLF1^tBRCT^ interaction with the C-terminal region of RAD18 is phosphorylation-dependent; the interaction was shown to be totally abolished when both S442 and S444 of RAD18 were mutated to alanine residues (13,37). To explore the structural mechanism of this phosphorylation-dependent interaction between SLF1 and RAD18, we produced and purified a His-MBP-tagged C-terminal construct of RAD18, denoted RAD18^CT^ (residues 423-495, Figure 3A, B). We also produced a mutant containing the point mutations S442D and S444D (RAD18^CT_S442D/S444D^), to mimic phosphorylation of these two serine residues. The results of analytical size exclusion chromatography (SEC) experiments indicated complex formation between SLF1^tBRCT^ and RAD18^CT_S442D/S444D^, but not with RAD18^CT^ (Figure 3C). Our results show that without phosphorylation at S442 and/or S444, the C-terminus of RAD18 cannot form a stable interaction with SLF1 in solution.

**Figure 4.**
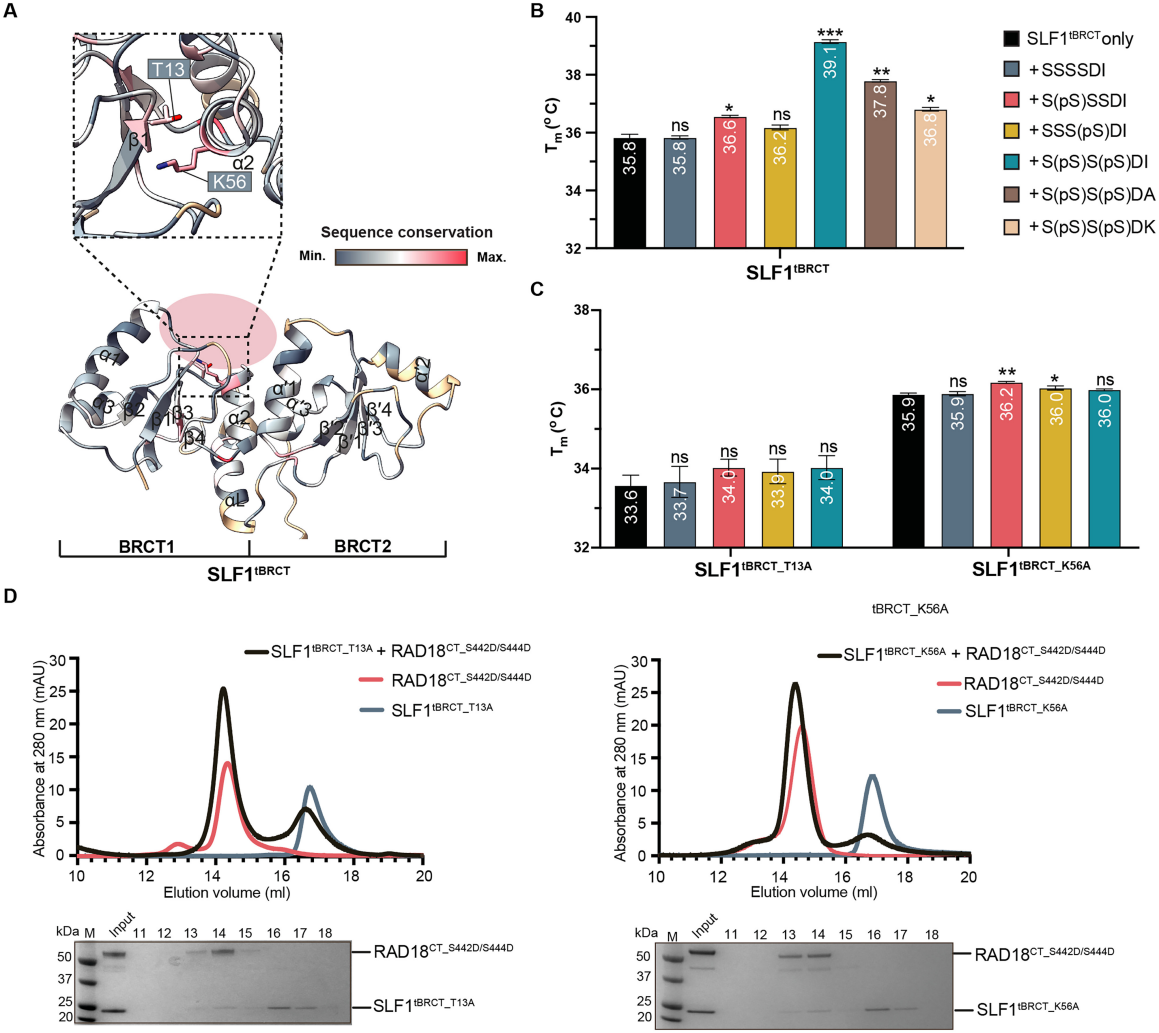
RAD18 phosphate recognition by SLF1 involves conserved phosphate-binding residues, T13 and K56. (**A**) AF2-predicted structure of SLF1^tBRCT^ highlighting conserved phosphate-binding residues (shown as sticks). Colouring is based on residue conservation obtained from a Chimera-generated structure-based sequence alignment for known tBRCT domains. Structural elements for the two BRCT domains (BRCT1, BRCT2) are labelled. (**B**) Summary of thermal melt derived melting temperatures for SLF1^tBRCT^ with chemically synthesized RAD18 peptides (see Figure 3A). *T*_m_ values for each condition are indicated. Error bars represent S.E.M from 3 separate experiments *P ≤ 0.05, **P ≤ 0.01, ****P ≤ 0.0001, ns = no significant difference for one-way ANOVA and post-hoc Dunnett T3 test comparing to SLF1^tBRCT^ only *T*_m_. (**C**) Summary of thermal melt derived melting temperatures for SLF1^tBRCT^ mutants, SLF1^tBRCT_T13A^ and SLF1^tBRCT_K56A^ with chemically synthesized RAD18 peptides containing phosphorylated S442 and/or S444. (**D**) Gel filtration for T13A (left) or K56A (right) mutant SLF1^tBRCT^ with phosphomimetic (S442D/S444D) RAD18^CT^. Coomassie-stained analysis of elution fractions are shown beneath each chromatogram. Data are representative of at least two repeats.

We then explored which residues of SLF1^tBRCT^ are important for interacting with phosphorylated RAD18. tBRCT domains, comprised of two tightly packed adjacent BRCT domains, function as key reader domains within the DDR, and are typically involved in the recognition of phosphorylated targets to transduce DNA damage signals initiated by kinases (74–76). Such domains are also present in key DDR proteins: BRCA1, BARD1, MCPH1, MDC1, PTIP, TOPBP1, 53BP1, and Nibrin. A conserved ‘two-anchor’ mode of phospho-target recognition has been elucidated from multiple structures of tBRCT domains in complex with phosphorylated peptides from their respective targets (74,76–79). A phosphorylated serine or threonine p(S/T) (‘anchor 1’, position 0) interacts with conserved phosphate-binding residues in the first BRCT domain, while tBRCT domains typically recognize a + 3-residue (‘anchor 2’) using a hydrophobic cleft at the interface of the two BRCT domains. The AlphaFold-predicted structure of SLF1 shows a typical tBRCT structure within its N-terminus, in which two BRCT domains (BRCT1 and BRCT2) are held together in a head-to-tail manner connected by a linker helix, αL (Figure 4A). The globular structure of each BRCT domain has a βαββαβα topology, forming a four-stranded parallel β-sheet surrounded by three α-helices, in which helices α1 and α3 are located on one side of the β-sheet with α2 on the other. A structure-based alignment for SLF1^tBRCT^ with all known human tBRCT domains, listed above, revealed two highly conserved and solvent accessible residues, T13 and K56 (Figure 4A). These two residues are located within β1 and α2 of BRCT1, forming a positively charged binding pocket. This finding suggested a conserved phosphate-binding function of SLF1 and led us to our hypothesis that phosphorylated RAD18 may interact with SLF1^tBRCT^ in a manner similar to other tBRCT domains.

The ‘two-anchor’ binding mode between other tBRCT domains and their target proteins involves both BRCT domains and can induce subtle movements of the BRCT domains (76), potentially stabilizing the overall fold. Using this rationale, we used synthesized RAD18 hexapeptides, with or without phosphorylation at S442 and/or S444, to carry out thermal melt experiments to test our hypothesis that a conserved mode of phosphate recognition mediates the observed complex formation between SLF1^tBRCT^ and phosphorylated RAD18. Compared to SLF1^tBRCT^ alone, incubation of SLF1^tBRCT^ with a double phosphorylated RAD18 peptide resulted in a significant increase in the melting temperature of SLF1^tBRCT^ (Δ*T*_m_ = 3.7°C) (Figure 4B, Supplementary Figure S5), supporting our earlier observations of a phosphorylation-dependent interaction. A RAD18 peptide containing single phosphorylation of S442 mediated a 0.8°C increase in the *T*_m_ of SLF1^tBRCT^, while single phosphorylation at S444 mediated a 0.4°C increase in *T*_m_. This suggests a more important role for pS442 compared with pS444 for stabilizing SLF1 through interaction.

To test whether phosphate recognition involves a conserved phosphate-binding pocket, we produced SLF1^tBRCT^ containing a point mutation at T13 or K56, SLF1^tBRCT_T13A^ and SLF1^tBRCT_K56A^, respectively. Mutation of either T13 or K56 of SLF1^tBRCT^ abolished the large increase in *T*_m_ observed in the presence of the double phosphorylated RAD18 peptide, suggesting the loss of phosphate binding in these mutants (Figure 4C). Compared to the wild-type protein, SLF1^tBRCT_T13A^ also displays a reduced thermal stability, suggesting that this residue contributes to the stabilization of the tertiary structure. CD analysis of these purified mutants indicated no change in secondary structure elements compared to wild-type SLF1^tBRCT^ suggesting that the observations made were not a consequence of misfolded protein (Supplementary Figure S3B). We also tested whether these mutations disrupt the complex formation observed between SLF1^tBRCT^ and RAD18^CT_S442D/S444D^ using SEC. These two mutations largely abrogated the previously observed shift in the RAD18^CT_S442D/S444D^ elution peak observed using wild-type SLF1^tBRCT^ (Figure 4D).

Together, our results indicate that double phosphorylation at S442 and S444 significantly enhances the interaction between RAD18 and SLF1^tBRCT^. Notably, crystal structures of SLF1^tBRCT^ in complex with two phosphorylated RAD18 peptides, released during the preparation of this manuscript, support these findings (47). Our results validate a functional role for both T13 and K56 of SLF1 in phosphorylated RAD18 recognition. Single phosphorylation of RAD18, or mutation of conserved phosphate-binding residues in SLF1^tBRCT^ results in the loss of stabilization of SLF1^tBRCT^ by double phosphorylated RAD18.

### Three hydrophobic residues contribute to stable complex formation between phosphorylated RAD18 and SLF1^tBRCT^

**Figure 5.**
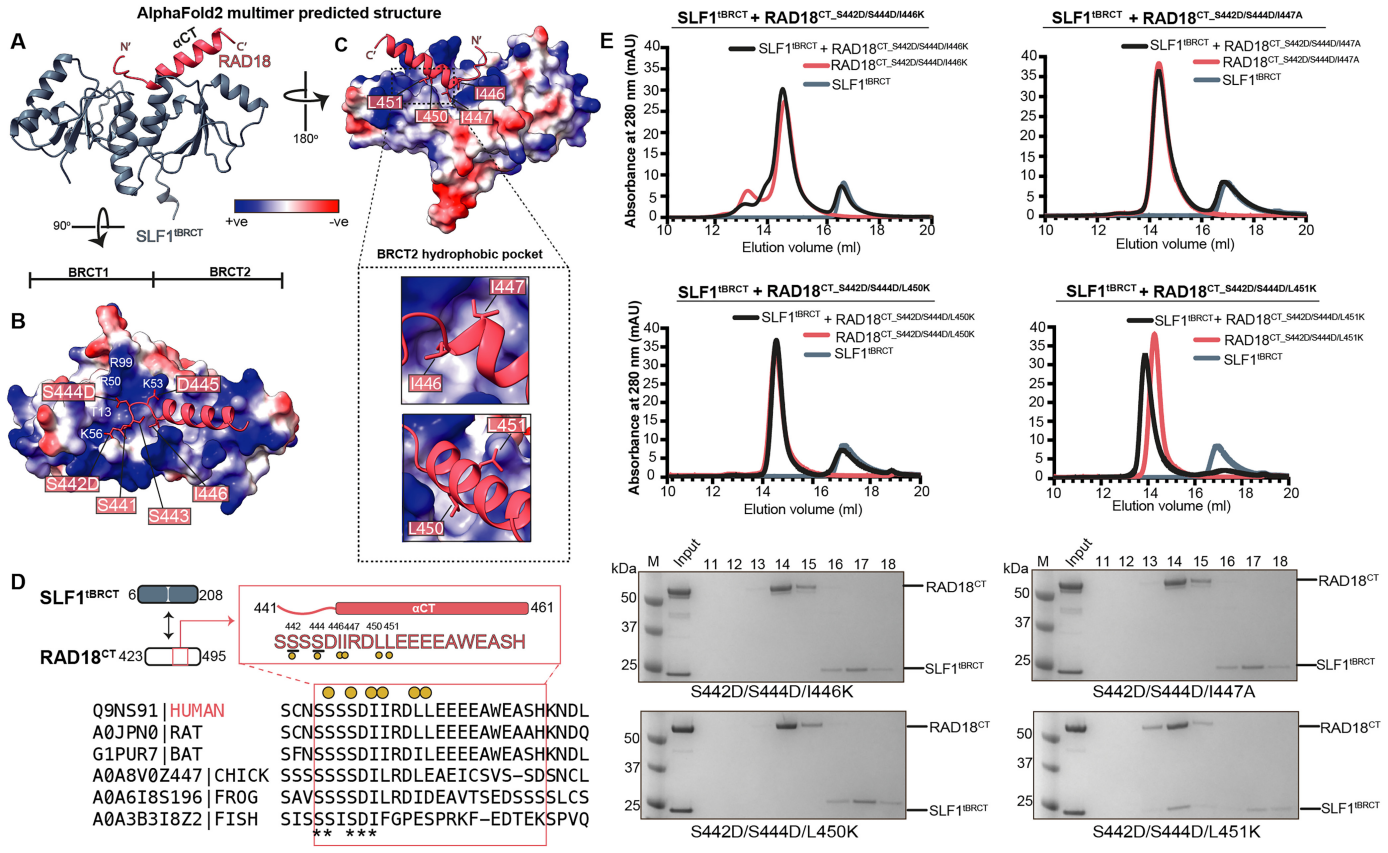
RAD18 hydrophobic residues are required for SLF1 binding. (**A**) AF2-predicted model for a RAD18^CT_S442D_S444D^-SLF1^tBRCT^ complex. SLF1 is shown in grey. RAD18 (residues 441–462, which were modelled with high pLDDT score) is shown in red. (**B, C**) The electrostatic potential surface of SLF1^tBRCT^ revealing an extensive basic binding surface and hydrophobic pocket accommodating the phosphomimetic (S442D/S444D) RAD18 C-terminus. The predicted positioning of the side chains of S441 to L451 are shown. Polar SLF1 residues within the interface are labelled in B). Hydrophobic side chains of RAD18 are highlighted in C). (**D**) Sequence alignment of RAD18 across species indicating evolutionary conserved residues within the C-terminal region, indicated by an asterisk (*). The residues tested for their functional role in SLF1 binding are indicated, with phosphorylation sites underlined. The relative position of this region within the RAD18^CT^ construct is illustrated in the schematic above. (**E**) Gel filtration for SLF1^tBRCT^ with phosphomimetic (S442D/S444D) RAD18^CT^ containing additional mutation of I446K, I447A, L450K, or L451K. Coomassie-stained analysis of elution fractions are shown beneath the chromatograms. Data are representative of at least two repeats.

To provide further structural insight into the complex formation observed in Figure 3, and the mechanistic basis for recognition of phosphorylated RAD18 by SLF1, we used AF2-multimer to predict the structure of SLF1^tBRCT^ with RAD18^CT_S442D_S444D^ (Figure 5A, Supplementary Figure S6). The model predicted that residues S441 to I446 of RAD18 span the two BRCT domains of SLF1^tBRCT^, consistent with the significant stabilization observed from thermal melt experiments using a short peptide corresponding to these residues (Figure 4B). In this model, the side chain of S442D of RAD18 acts as ‘anchor 1’, anchored by the conserved pocket formed between β1 and α2 containing T13 and K56 in the BRCT1 of SLF1^tBRCT^, closely resembling the location of a phosphate moiety observed in previous crystal structures of phosphopeptide-bound tBRCT domains (74,76–79). While S441 and S443 make no direct contact with SLF1^tBRCT^, S444D and D445 form further ionic interactions with a positive surface formed by R50, R99, and K53 in BRCT1 (Figure 5B). The recent structural evidence discussed above supports this prediction. Superimposition with our AF2 model shows that the conformation of the short peptide used in their study closely aligns with that predicted for our longer RAD18^CT^ construct (Supplementary Figure S6D, E). This structural alignment reinforces the validity of our model.

Residue I446 (position + 4 relative to S442D) was predicted to be the first residue of an α-helix within the RAD18 C-terminal region (residues 446 to 461, labelled as αCT) (Figure 5A). The N-terminus of this RAD18 helix appears to be stabilized by hydrophobic interactions with residues at the interface of the two SLF1 BRCT domains, analogous to the typical tandem BRCT recognition of ‘anchor 2’, although mediated by a group of hydrophobic residues as opposed to a single residue. Residues I446, I447, L450, and L451 were all predicted to occupy hydrophobic pockets at the BRCT1/2 interface or within the second BRCT domain (Figure 5C). This anionic helix fits within a channel formed by the second BRCT domain, suggesting the specificity of SLF1 for recognition of RAD18. Sequence alignment of the C-terminal RAD18 region showed high conservation of the two critical phosphorylation sites, S442 and S444, as well as I446. In comparison, residues I447, L450, and L451 show less conservation across species (Figure 5D). Using thermal melt experiments, we found that mutation of I446 within our double-phosphorylated hexapeptide abrogated the thermal stabilization of SLF1^tBRCT^ (Figure 4B). An I446K mutation had the most significant impact on thermal stabilization, reducing the *T*_m_ by 2.3°C relative to the double-phosphorylated peptide, highlighting a strong preference for a hydrophobic residue at this position. An I446A mutation led to a 1.3°C decrease in *T*_m_, presumably due to the loss of hydrophobic contacts from the shorter side chain. These observations suggest that the stabilizing effect of the phosphorylated peptide above is sequence-specific, with a preference for an isoleucine residue positioned +4 from pS442. However, further investigation is needed to determine how specific the binding of SLF1^tBRCT^ to RAD18 is compared to other di-phosphorylated peptides.

To further experimentally validate the observations from the AF2 model, and to ascertain the contribution of the other hydrophobic residues for SLF1^tBRCT^ binding, we individually mutated I446, I447, L450, and L451 within the RAD18^CT_S442D/S444D^ construct. Although the contribution of these hydrophobic residues has recently been explored using isothermal titration calorimetry (ITC) experiments with double isoleucine and leucine mutant RAD18 peptides (47), the contribution of individual residues on the interaction of the full C-terminus of RAD18 with SLF1 has not been tested. Our attempts to purify I447K consistently yielded a predominantly cleaved protein. However, we successfully purified an alanine mutant, I447A. Using SEC, we found that I446K, I447A, and L450K, but not L451K disrupted the stable interaction between SLF1^tBRCT−^RAD18^CT_S442D/S444D^ (Figure 5E). These results indicate that three hydrophobic residues in the C-terminus of RAD18 function as a critical anchor for stable complex formation with SLF1. This supports a model in which SLF1 employs an atypical two-anchor interaction mechanism for recognition of phosphorylated RAD18.

### SLF1^tBRCT^ displays high-affinity DNA binding via a shared binding site with phosphorylated RAD18

**Figure 6.**
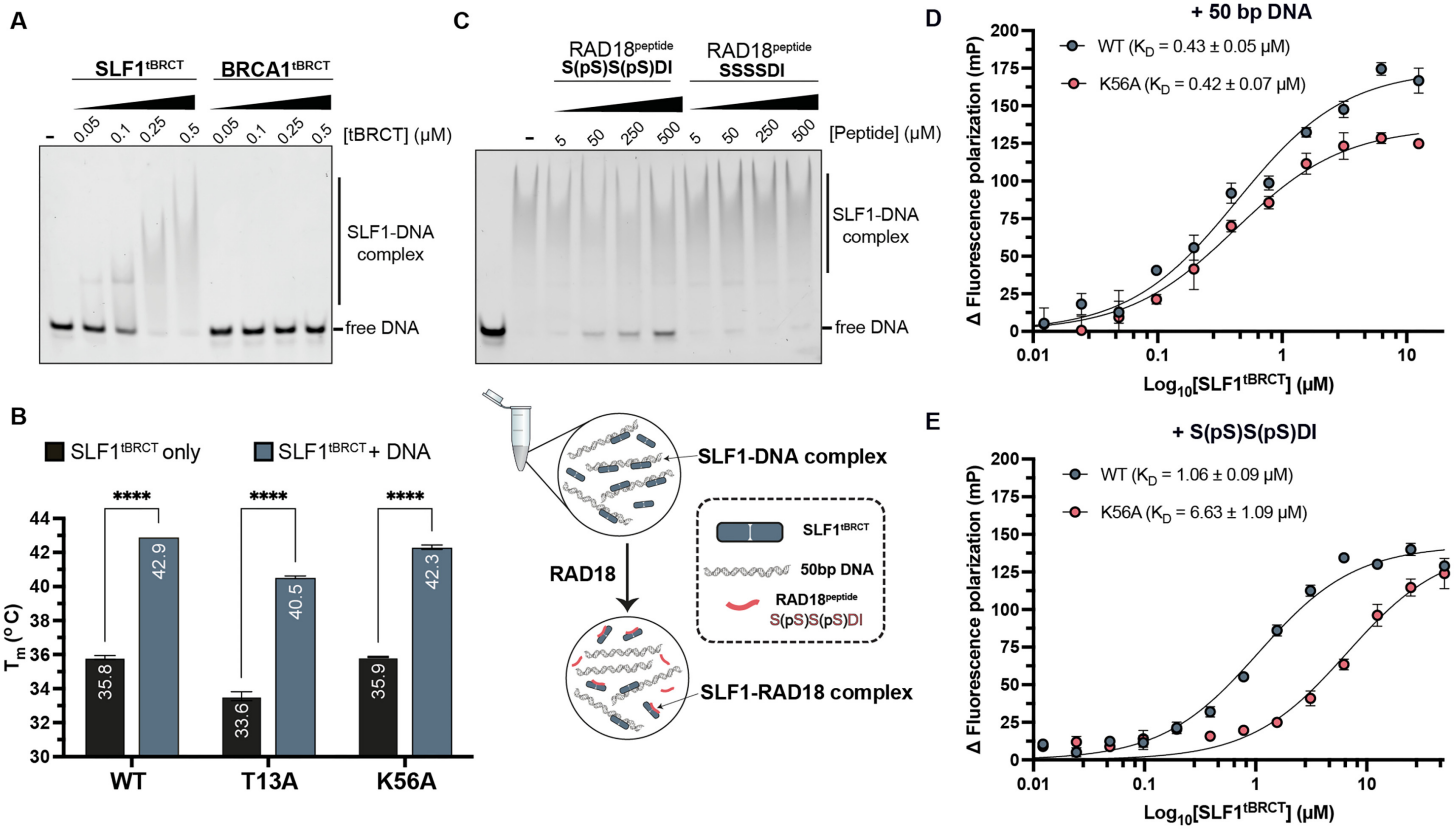
DNA and phosphorylated RAD18 share a common SLF1 interaction surface. (**A**) EMSA gel for the interaction of SLF1^tBRCT^ or BRCA1^tBRCT^ with 6-FAM labelled 50 bp DNA (representative of 3 replicates). Each lane contains 10 nM DNA, with the concentration of protein indicated. (**B**) Summary of thermal melt derived melting temperatures for WT, T13A and K56A mutant SLF1^tBRCT^ proteins with 50 bp DNA. *T*_m_ values for each condition are labelled. Error bars represent SEM from three separate experiments. ****P ≤ 0.0001 for unpaired, two-tailed *t*-test for the *T*_m_ of each SLF1^tBRCT^ protein with or without DNA. (**C**) Competition EMSA indicating competition of double phosphorylated RAD18 peptide (S(pS)S(pS)DI) for DNA binding to SLF1^tBRCT^ (representative of three replicates). Each lane contains 10nM DNA, with the concentration of SLF1^tBRCT^ protein and RAD18 peptide indicated. A schematic illustrating the assay setup and observations of the EMSA are illustrated in the panel below. (**D, E**) Fluorescence polarization assay for interaction between 6-FAM labelled 50 bp DNA (D) or 6-FAM labelled S(pS)S(pS)DI peptide (E) with WT or K56A mutant SLF1^tBRCT^. Error bars represent SEM (*n* = 3). Binding affinity (*K*_D_) determined from non-linear fitting of the data is indicated.

The AF2-predicted RAD18 binding interface of SLF1^tBRCT^ displays a highly positive surface charge (Figure 5B). Comparison of the surface charge properties for tandem BRCT domains reveals that the surface of SLF1^tBRCT^ is much more cationic than the tBRCTs found in other DDR proteins, such as BRCA1^tBRCT^. This extensive positive surface charge of SLF1 may explain how this protein is adapted for recognition of the basic RAD18 C-terminal sequence. Given SLF1’s assumed role as a DNA damage recognition factor, we hypothesized that this enrichment of positively charged side chains may also confer an intrinsic DNA-binding property to facilitate interactions with DNA at sites of replication fork stalling. To explore this, we used a 6-FAM labelled 50 bp DNA substrate to test for an SLF1-DNA interaction using EMSAs. Our results revealed a high affinity of SLF1^tBRCT^ towards the 50 bp substrate, potentially with multiple SLF1^tBRCT^ molecules binding to the DNA, as indicated by the observed smearing of DNA bands with slower mobility (Figure 6A). In comparison, we observed no interaction between DNA and BRCA1^tBRCT^. This difference in DNA binding activity is likely attributable to the differences in electrostatic surface properties, suggesting a unique property of SLF1^tBRCT^ conferred by its distinctive surface.

Given the strong DNA binding affinity towards 50 bp DNA, we also tested the binding of SLF1^tBRCT^ to nucleosomes. Using 1 μM SLF1^tBRCT^, we observed a significant shift in migration (Figure 2B). This observed affinity was lower compared to that observed with 50 bp DNA, possibly due to reduced accessibility to the packaged DNA within the nucleosome. However, the affinity was higher than that of ARD binding. We also noted a much-reduced migration, suggesting the formation of multiple protein complexes, and thus the interaction of multiple SLF1^tBRCT^ molecules with each nucleosome. In EMSA experiments using both SLF1^ARD^ and SLF1^tBRCT^ (Figure 2B, bottom panel), we observed a super shift of these SLF1^tBRCT^-bound nucleosome complexes at concentrations of SLF1^ARD^ at which nucleosome binding had been observed previously. The small shift in migration was consistent with the additional binding of SLF1^ARD^ to SLF1^tBRCT^-bound nucleosome. SEC analysis indicated no interaction between SLF1^ARD^ and SLF1^tBRCT^ in solution (Figure 2D). Together with our EMSA results, this suggested that the purified domains themselves do not facilitate each other's binding, but instead seem to recognize separate features of the nucleosome.

To further explore the interaction of SLF1^tBRCT^ with DNA, we measured the thermal stability of SLF1^tBRCT^ in the presence of the 50 bp DNA substrate (Figure 6B). Here, we observed a significant increase in the melting temperature of SLF1^tBRCT^ (Δ*T*_m_ = 7.1°C) in the presence of 50 bp DNA. A similar thermal-stabilization effect was observed for the T13A (Δ*T*_m_ = 6.9°C) and K56A (Δ*T*_m_ = 6.4°C) mutants. The *T*_m_ increase was much greater than that observed with the double phosphorylated RAD18 peptide (Figure 4B, C). These results indicate a stabilizing effect of DNA binding on the SLF1^tBRCT^ tertiary structure, with this DNA substrate likely able to interact with a larger area/more surface residues across both BRCT domains than the phosphorylated RAD18 hexapeptide. We then performed competition EMSAs using SLF1^tBRCT^ in the presence of 50 bp DNA and increasing concentrations of RAD18 peptides. The results show partial competition of the DNA substrate by the double phosphorylated RAD18 peptide, but not by the unphosphorylated peptide (Figure 6C). Together, these results indicate that SLF1^tBRCT^ has both phospho-target and DNA-binding functions, and point towards a shared binding surface of the two substrates. Consistently, an AF3 predicted model of SLF1^tBRCT^ with this 50 bp DNA substrate also predicts DNA to interact with the same surface as the RAD18 peptide (Supplementary Figure S7A).

To further explore the competition between DNA and phosphorylated RAD18 for SLF1^tBRCT^ binding, we used fluorescence polarization to compare the affinity for our 50 bp substrate and a 6-FAM labelled version of our double phosphorylated RAD18 hexapeptide. The binding affinity (K_D_) of SLF1^tBRCT^ towards 50 bp DNA was calculated to be 430 nM (Figure 6D), consistent with observations of the EMSA (Figure 6A). In comparison, the binding affinity for the 6-FAM-labelled RAD18 peptide was approximately 1 μM (Figure 6E). Given that this short peptide comprises only six residues of the RAD18 C-terminus, this represents a relatively high affinity compared to the DNA binding. Notably, the K56A mutation significantly reduced the binding affinity for the phosphorylated peptide (*K*_D_ = 6.63 μM) but had no significant effect on the affinity towards DNA, consistent with the interaction with DNA involving a broader surface of negatively charged surface residues (Figure 6D, E, Supplementary Figure S7A).

## Discussion

A comprehensive understanding of protein interactions involved in replication-dependent repair mechanisms is highly relevant to fundamental cancer biology, to better predict treatment outcomes and identify novel therapeutic targets and strategies. Our study focuses on SLF1, a multi-domain DDR protein that emerged as an essential component of the RAD18–SLF1–SLF2–SMC5/6 pathway which accumulates at stalled replication forks to facilitate efficient replication-coupled DNA repair (13). We provide new structural evidence for the interaction of SLF1 with the unmethylated tail of histone H4, as well as insights into its specific interaction with phosphorylated RAD18, and a high-affinity DNA-binding property within the same domain involved in RAD18 recognition. These findings provide an insight into the molecular mechanism by which DDR signals at stalled replication forks are propagated through post-translational modifications and protein/DNA interactions of SLF1. The presence of domains that recognize both a histone marker of the chromatin replicative state and damage-dependent signals enables SLF1 to bridge DNA repair and DNA replication, thereby integrating these critical processes.

Our crystal structure provides direct evidence that SLF1^ARD^ recognizes the histone H4 N-terminal tail containing unmethylated K20 (H4K20me0), a signature of post-replicative chromatin (33,34). The interface between SLF1 and H4 closely resembles the interface of H4 with TONSL or BARD1. This conserved interface involves acidic residues that form the concave surface, and ‘fingers’ of ANKs 1–3. A conserved tryptophan also plays a crucial role in anchoring the H18 residue of H4 through stacking interactions and contributes hydrophobic and polar contacts to the interface. Specificity for H4K20me0 is provided by an acidic channel in which K20 establishes strong polar contacts with the side chains of E847, E808, and D838 in SLF1, that would be disrupted by methylation at K20. This ARD-nucleosome interaction provides a mechanism of temporal restriction, ensuring that these post-replication repair complexes accumulate during the S/G2 phases of the cell cycle to repair DNA damage present in replicating/replicated chromatin. The ARD predominantly mediates interactions with H4 residues R17, H18, R19, and K20, which are part of a basic stretch of the tail known to mediate chromatin compaction through interaction with the acidic patch on neighbouring nucleosomes (33,80). Thus, protein binding to this position may also influence chromatin structure, preventing chromatin compaction when DNA damage occurs.

Recent cryo-EM structures of BARD1-bound nucleosome revealed that the ARD residues K423, H426, and R427 located in Finger 0 of BARD1 are in close vicinity to the phosphate-backbone of DNA (35,36). Similar residues in SLF1 (T803, N804, and K805) can be identified in a structurally conserved position of Finger 0 (Supplementary Figure S2C). Given that we were able to observe a shift in the electrophoretic mobility of unmodified nucleosome using SLF1^ARD_4A^ at 25 μM (Supplementary Figure S4), it is plausible that these residues may contribute to additional electrostatic interactions that aid in ARD-H4K20me0 binding to stabilize the interaction. Within the SLF1 protein, the ARD is situated within an Nse5-like structured domain at the C-terminal region, separated by a flexible linker from the rest of the ‘Nse5-like’ domain. The core Nse5-like structure is predicted to interact with the ‘Nse6-like’, or CANIN domain, of SLF2 (30,81). Further structural studies are required to elucidate whether other regions within this SLF1-SLF2 complex, beyond the ARD, establish additional contacts with the nucleosome that may further stabilize the interaction.

The role of tandem BRCT repeats in recognizing DNA damage-induced phosphorylation motifs is well established. While the precise timing and kinase responsible for RAD18 phosphorylation at S442 and S444 remain elusive, a phosphorylation-dependent interaction involving these residues with SLF1^tBRCT^ is crucial for efficient replication-coupled DNA repair (13,37). Our results, supported by a recent crystal structure released during the preparation of this manuscript, support a canonical tBRCT phosphate-recognition mechanism involving SLF1 residues T13 and K56 (47). Our thermal melt experiments indicated that phosphorylation of S442 led to a more substantial increase in thermal stability compared to phosphorylation at S444, with a synergistic effect observed when both positions are phosphorylated. This aligns with the recent structural data showing that pS442 is fully buried within the interface, while pS444 is predominantly solvent exposed. The negative charge of phosphorylated S444 likely further strengthens the interaction with the positive surface of BRCT1 of SLF1. Typically, phosphorylated motifs of characterized binding partners of other tandem BRCT domains are located within intrinsically disordered regions. The first anchor is usually a pS/T (consistent with pS442 for RAD18), and ‘anchor 2″ typically comprises a single hydrophobic residue at the +3 position relative to ‘anchor 1’ (76,82,83). Compared to these observations, our AF2 model and structure-based mutagenesis, alongside recent structural data, suggest a more extensive ‘second anchor’. Our results demonstrate that three hydrophobic residues (I446, I447, and L450) significantly contribute to complex formation. The crystal structure (47) and our AF2 model also suggest that these residues are within the N-terminus of an alpha-helix that sits across the second BRCT domain. Thus, RAD18 appears to interact more extensively with SLF1 than other phosphorylated targets with their respective tBRCT-containing proteins.

The diverse surface properties among tandem BRCT domains, driven by their low sequence conservation, can facilitate specificity for a wide range of binding partners and unique functional activities outside of phosphate recognition. Unlike the BRCA1^tBRCT^, we revealed a high affinity DNA-binding property of SLF1^tBRCT^, likely attributable to its extensive cationic surface. Our results suggest that the phosphate-binding pocket of SLF1^tBRCT^ also serves as a binding site for nucleosomal or free DNA. SLF1’s unique DNA-binding property may regulate the interaction between the SMC5/6 complex and DNA, thereby modulating the function of the SMC5/6 complex in DDR. Moreover, its dual binding to DNA and phosphorylated RAD18 at a shared site could aid localization to stalled replication forks, facilitating rapid stabilization at damage sites in response to damage-induced ubiquitination and competition by RAD18. Notably, while the calculated affinities of SLF1^tBRCT^ for a 50 bp substrate and RAD18 peptide were approximately 430 nM and 1.06 μM, respectively, in our assays, recent ITC experiments with a RAD18 peptide (residues 437–452) reported a much higher affinity of 12 nM (47). This discrepancy in affinity measurement, particularly with respect to the RAD18 peptide, could arise from differences in the assay setup, the fluorophore on our short hexapeptide, or the use of a longer peptide, particularly the inclusion of additional hydrophobic residues that make up ‘anchor 2’ in their studies (47). Additionally, crystal structures revealed an additional interface with SLF1^tBRCT^ involving RAD18 residues N-terminal of S442 (47), while the C-terminal end of the RAD18 α-helix (residues 446–461), predicted by AF2, enriched in acidic amino acids, may establish further polar contacts with the uniquely basic surface residues of SLF1^tBRCT^. Consequently, full-length phosphorylated RAD18 may indeed effectively compete with DNA. The functional consequences of this competition would be interesting to explore further. Our fluorescence polarization assays revealed that the K56A mutant, which disrupts phosphorylated RAD18 interaction, minimally impacts the DNA-binding property. Thus, this mutation presents an opportunity to dissect the contributions of these two functions to DNA repair in future cell-based experiments.

Our research supports a role of SLF1 in recognizing damage-induced signals in post-replicative chromatin. The release of AlphaFold3 during the preparation of this manuscript allowed us to create a predicted model for the full-length SLF1 protein in complex with an unmodified nucleosome. This model supports our proposed mechanism whereby SLF1’s tBRCT domain binds to DNA, while its ARD domain binds to H4K20me0, stabilizing its interaction with nascent chromatin (Supplementary Figure S7B). However, further investigation is needed to fully elucidate how SLF1’s interaction with phosphorylated RAD18 contributes mechanistically to replication-coupled repair. Recruitment of RAD18, SLF1, SLF2 and SMC5/6 to interstrand cross-links relies on RNF8, MDC1, and RNF168, which introduce ubiquitination markers of DNA damage. RAD18’s UBZ domain recognizes such signals by binding to H2A ubiquitinated at K13/15 (84). Additionally, RNF168 signals G-quadruplex (G4) DNA structures, stabilized by CX5461, with SLF2 and SMC5/6 shown to support DNA replication in response to this inhibitor (85). Hence, RNF8/RNF168 ubiquitin-ligase activity may generally promote the enrichment of RAD18 and SLF1 at stalled replication forks or under replication stress, with RAD18 acting as an adaptor. The dual recognition of damage-dependent modifications and H4K20me0 on replicating chromatin by SLF1 shares similarities with the nucleosome recruitment mechanism of BRCA1 by BARD1. BARD1 uses its ARD and a BUDR (tandem BRCT-domain-associated ubiquitin-dependent recruitment) motif within its tBRCT to recognize H4K20me0 and H2AK15ub marks, respectively, with inter-domain interactions involved in stabilizing the binding to the nucleosome (35,86). Unlike BARD1, where the tBRCT and ARD are adjacent, SLF1’s tBRCT and ARD domains are structurally distant. Although we did not observe inter-domain interactions between the isolated domains of SLF1 (Figure 2D), it is plausible that upon interaction with nucleosomes, these domains might establish additional contacts with each other, RAD18, or the nucleosome. The structural arrangement of domains in SLF1 also offers conformational flexibility which may enable it to interact simultaneously with neighbouring nucleosomes. For example, SLF1’s tBRCT may engage phosphorylated RAD18 bound to H2AK15ub on one nucleosome, while its ARD associates with H4K20me0 on a neighbouring, newly deposited nucleosome. This could facilitate RAD18 recruitment in post-replicative chromatin or enable competition with other DNA repair proteins in a chromatin environment retaining partial H4K20me2, as discussed in recent publications (87,88).

In summary, our research provides a structural understanding of the SLF1 interaction network, revealing the roles played by its tandem BRCT and ARD domains. Continued research efforts to fully elucidate the dynamic nature of these interactions and their contribution to the DDR will further enhance our comprehension of SLF1’s function within the RAD18-SLF1-SLF2-SMC5/6 pathway during replication-coupled repair. Given the significance of these domains across other DDR proteins, these structural insights may also pave the way for potential drug-targeting strategies aimed at modulating the activities of SLF1, as well as the tBRCT and ARD domains involved in DNA damage repair pathways more broadly.

## Supplementary Material

gkae831_Supplemental_File

## Data Availability

The crystal structure of SLF1-H4 was deposited in Protein Data Bank with code: 8PEF.

## Notes

Present address: Nazia Nasir, Research and Innovation Service, University of Leeds, Nexus, Discovery Way, Leeds LS2 3AA, UK.

Present address: Michael Jenkyn-Bedford, The Rockefeller University, 1230 York Avenue, New York, NY 10065, USA.
