# Supplementary material for: Structural mechanisms of SLF1 interactions with Histone H4 and RAD18 at the stalled replication fork
Source: Nucleic Acids Res. 2024 Oct 3;52(20):12405–21. doi: 10.1093/nar/gkae831 (PMC11551741; doi:10.1093/nar/gkae831)

Supplementary Figures

Supplementary Figure S1: Purified protein samples of SLF1, RAD18, BRCA1, and NCP
Analysis of approximately 2 µg of each purified domain (not already presented in Figure 3B) used in this study. Protein samples were separated by SDS-PAGE using 4-12 % Bis-Tris gels. For nucleosome, 10 µg of sample was separated using a 16% Tricine gel.

Supplementary Figure S2: Structural analysis and comparison of the ARD structures from SLF1, TONSL and BARD1
A) Crystal structure of SLF1^{ARD}-H4^{peptide}:Omit Fo-Fc map (σ=2.0) is shown for the H4^{peptide}. **B)** Cartoon representation of SLF1^{ARD} (in grey) in complex with H4^{peptide} (in yellow), shown from above to highlight the individual helices of each ankyrin repeat (labelled ANK1-4) and the extended β-loop structures, referred to as “fingers”. **C)** Sequence alignment of the SLF1^{ARD} (residues 802-934), BARD1^{ARD} (residues 422-546), and TONSL^{ARD} (residues 523-669). The corresponding structural elements formed by each domain are depicted below the aligned residues. **D)** The sequence alignment in C) is visually represented by the colour rendering of the SLF1^{ARD} surface according to sequence conservation using ChimeraX. The conserved H4K20me0 recognition surface is outlined using a dashed line. **E)** H4K20me0 interacting residues are structurally conserved in SLF1, BARD1 and TONSL ARDs. Superimposition of the ARD structures of SLF1 (grey), BARD1 (PDB: 7E8I) (35) (yellow) and TONSL (PDB: 5JA4) (red) (33). An enlarged view of the indicated region of conserved H4K20me0 binding residues is presented below.

Supplementary Figure S3: Circular dichroism spectra for WT and mutants of SLF1.
A) CD spectra for WT and 4A mutant SLF1^{ARD}. **B)** CD spectra for WT,T13A, and K56A mutants of SLF1^{tBRCT}. The average ellipticity recorded from three replicate measurements was converted to mean residue ellipticity and is plotted as a function of wavelength.

Supplementary Figure S4: Representative electrophoretic mobility shift assay comparing WT and 4A mutant SLF1^{ARD} interactions with unmodified nucleosome core particles (NCPs). The assay was performed using up to 50 µM of purified ARD proteins, with each lane containing 10 nM NCPs. The concentration of SLF1^{ARD} in each lane is indicated. The figure is representative of two independent replicates.

Supplementary Figure S5: Representative thermal melt curves for thermal stability assays
A-C) Representative melt curves for SLF1^{tBRCT}(**A**), SLF1^{tBRCT_T13A} (**B**), or SLF1^{tBRCT_K56A} (**C**), with chemically synthesised RAD18 peptides phosphorylated at different positions (pS). Reactions containing peptides only served as controls for non-specific signals from the substrate. **D-F)** Representative melt curves for SLF1^{tBRCT}(**D**), SLF1^{tBRCT_T13A} (**E**), or SLF1^{tBRCT_K56A} (**F**), in the presence of 50 bp DNA. Reactions containing the DNA substrate only served as a control for non-specific signals arising from the substrate. The observed melting temperature for the apo SLF1^{tBRCT} protein in each set of experiments is indicated for each panel.

Supplementary Figure S6: AF2 predicted models for SLF1^{tBRCT}-RAD18$^{CT_S442D/S444D}$ complex.
A) Superimposition of the five AF2-predicted structural models for the complex between SLF1^{tBRCT} (grey) and RAD18$^{CT_S442D/S444D}$ in cartoon representation. RAD18 from each model is shown in a different colour (top 1 ranked model is in red). Enlarged view of the interface is also shown to highlight the consistency of the predicted positions of side chains S442D, S444D, D445, and I446 across the models. **B)** The highest ranked AF2 model, with each residue coloured by pLDDT score. Residues 441-463 of RAD18 are modelled well (pLDDT score >70). **C)** The predicted alignment error (PAE) plot for the highest ranked AF2 model. Chain A is SLF1 (6-208), and chain B is RAD18 (423-495). **D)** Superimposition of our AF2-predicted SLF1^{tBRCT} -RAD18$^{CT_S442D/S444D}$ structural model with the crystal structure of SLF1^{tBRCT} with a phosphorylated RAD18 peptide (PDB: 8IR2 (47)) using ChimeraX. Only residues 441-463 of RAD18 in our AF2 model, which were confidently modelled, are presented. **E)** Representation of side chains of RAD18 residues S442-L451, within the superimposition, to highlight the highly similar conformation observed in both models.

Supplementary Figure S7: AF3 predicted SLF1 complexes.
A) Superimposition of the five Alphafold 3 (AF3)-predicted structural models for the complex between SLF1^{tBRCT} with our 50 bp DNA substrate shown in cartoon representation. The electrostatic potential surface of SLF1^{tBRCT} is shown, highlighting the highly basic surface property of this domain. An enlarged

view of the DNA interaction surface, predicted in all five AF3 models, is shown below, with the position of the phosphate binding pocket involved in phosphorylated RAD18 binding highlighted. **B)** The highest ranked AF3-predicted structural model for the complex between full-length SLF1 and unmodified nucleosome. Surface representation is used to highlight the positions of the tandem BRCT domains and ARD within SLF1. The tails of each histone protein are presented without surface representation for simplicity. The predicted alignment error (PAE) plots for the highest-ranked AF3 model for each predicted structure are shown below each model in panels A and B.

Supplementary Figure S1

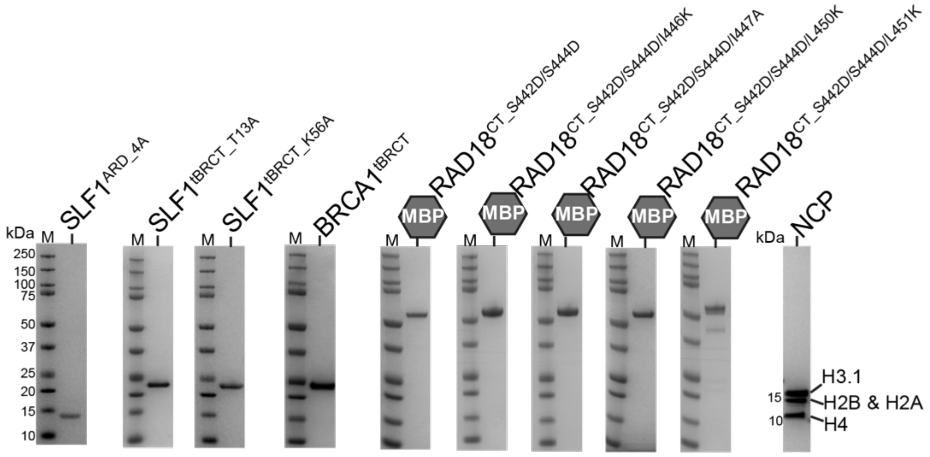

Supplementary Figure S2

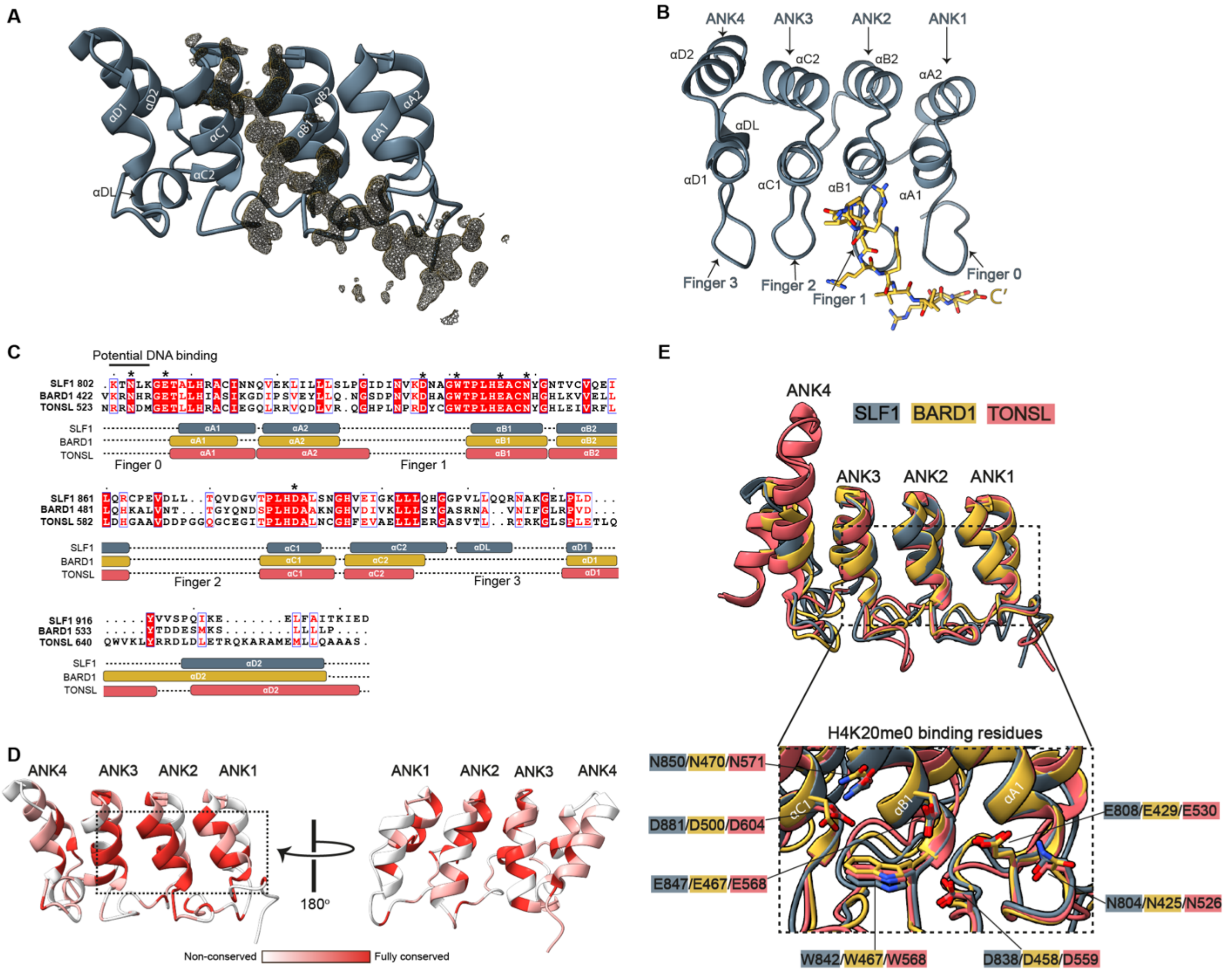

Supplementary Figure S3

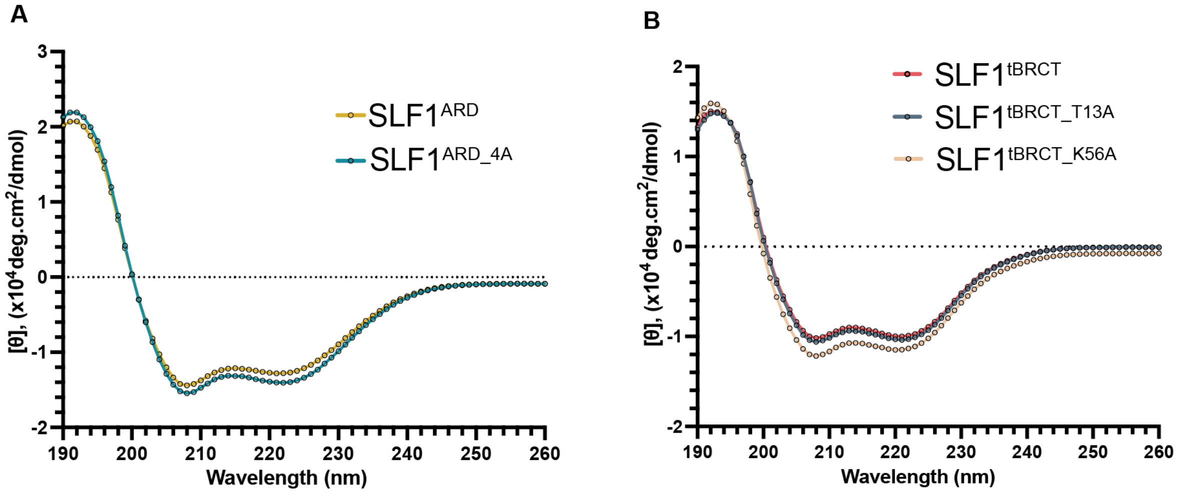

Supplementary Figure S4

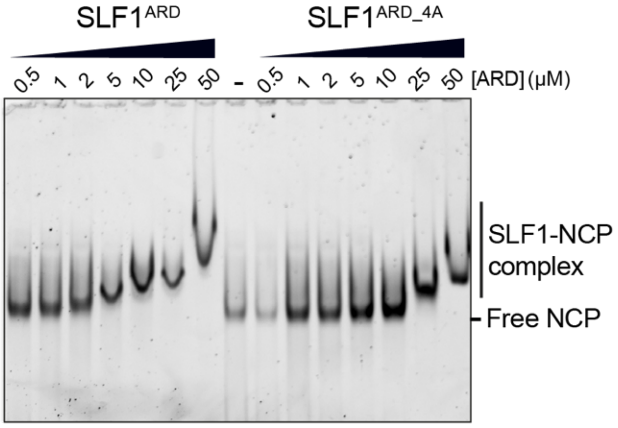

Supplementary Figure S5

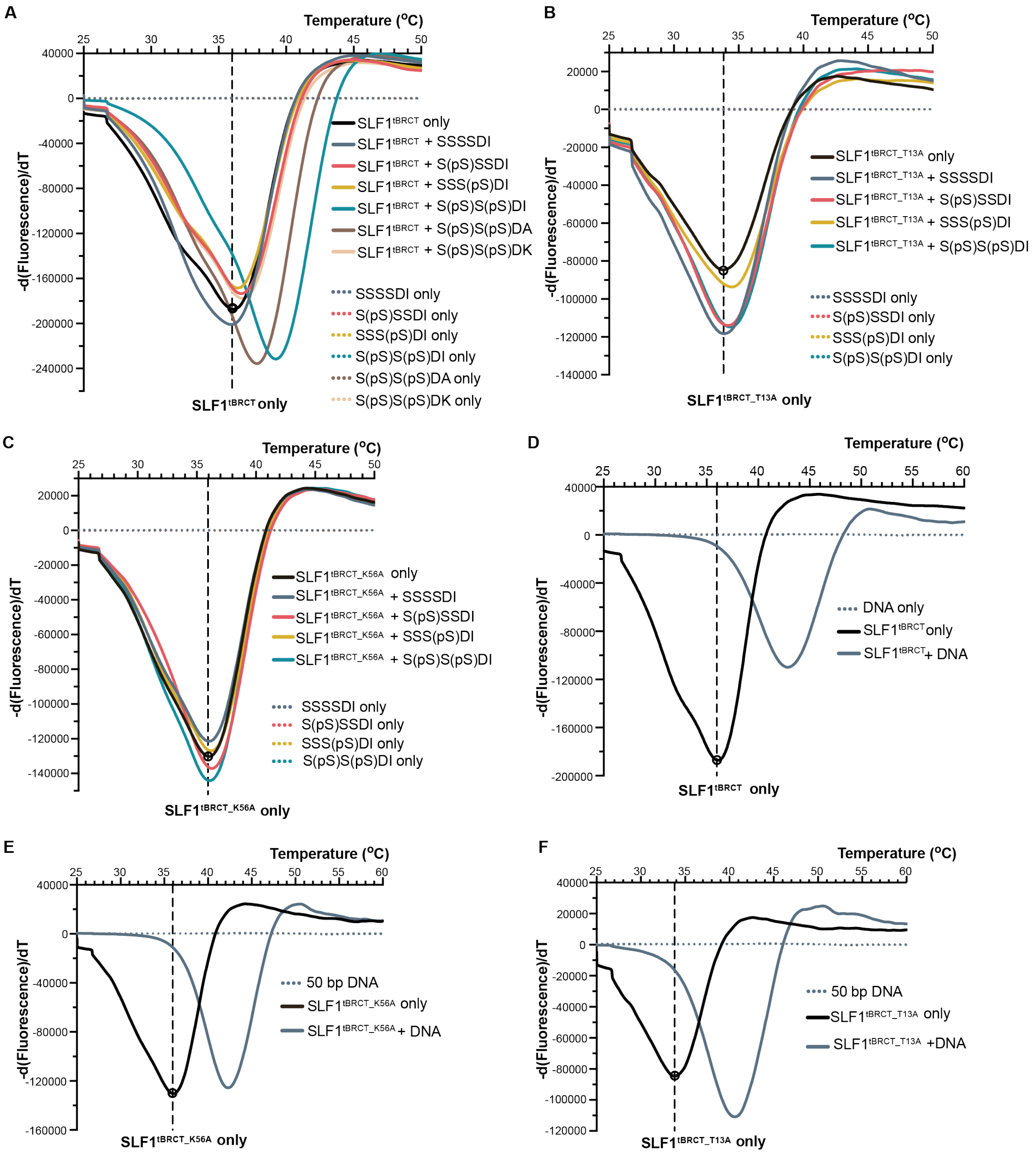

Supplementary Figure S6

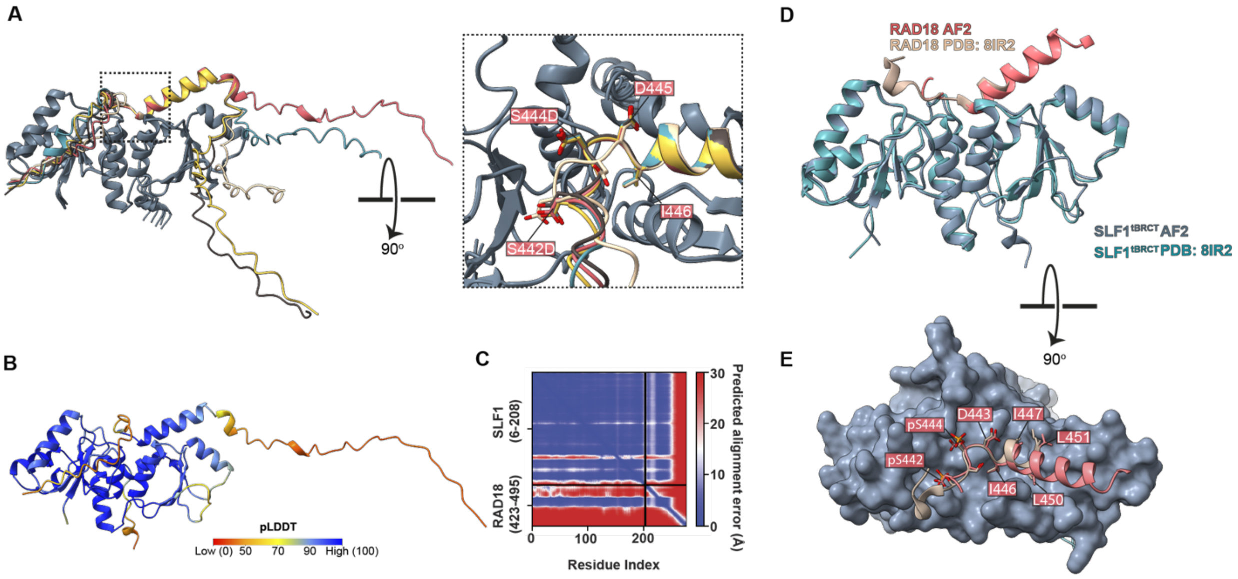

Supplementary Figure S7

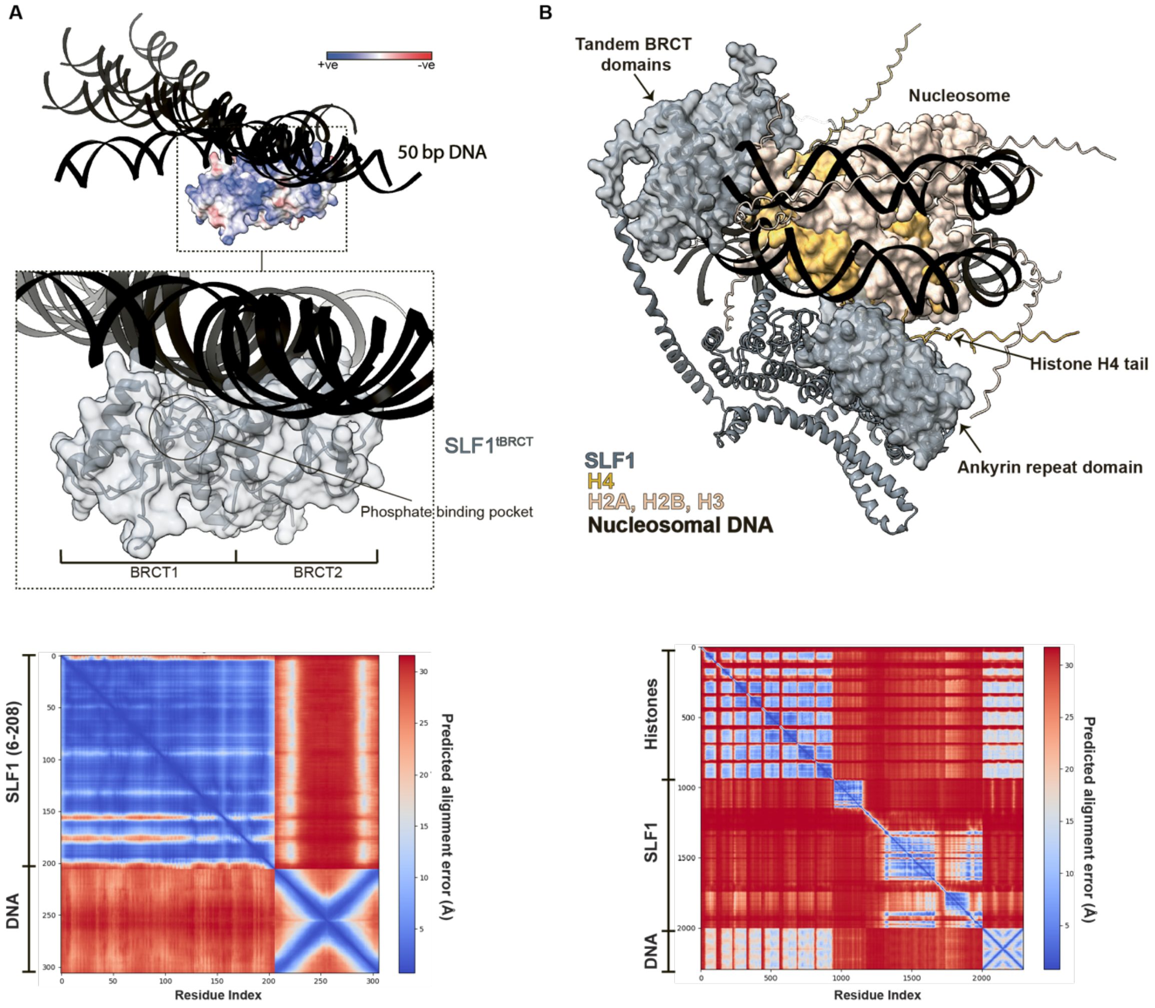

Supplement: gkae831_Supplemental_File [file gkae831_supplemental_file.pdf]
